# A meta-analysis of immunosuppressive and Pharmacological therapies in aplastic anaemia with and without Indigenous equine antithymocyte globulin (eATG)

**DOI:** 10.1007/s00277-026-06779-7

**Published:** 2026-01-22

**Authors:** Biju George, Cecil Reuben Ross, Sharat Damodar, Tulika Seth, Tuphan Kanti Dolai, Saswata Banerjee, Rajan Manguesh, Pankaj Malhotra

**Affiliations:** 1 Department of Hematology, Christian Medical College, Kilminnal, Vellore, Tamil Nadu India; 2 Medicine and Haematology Department, St. John’s Medical College, Bengaluru, Karnataka India; 3 Mazumdar Shaw Medical Centre, Narayana Health City, Bengaluru, Karnataka India; 4 Hematology Department, All India Institute of Medical Sciences, AIIMS, New Delhi, India; 5 Department of Haematology, Nil Ratan Sircar Medical College and Hospital, Kolkata, West Bengal India; 6 Bharat Serums and Vaccines Limited, Mumbai, India; 7 Department of Clinical Hematology & Medical Oncology, Postgraduate Institute of Medical Education & Research, Chandigarh, India

**Keywords:** Aplastic anaemia, Immunosuppressive therapy, Antithymocyte globulin, THYMOGAM, ATGAM, Cyclosporin, Eltrombopag

## Abstract

Aplastic anaemia (AA) is a rare bone marrow failure disorder with a notably higher incidence in Asian populations, including India. Due to limited access to bone marrow transplantation (BMT) in resource-limited settings, immunosuppressive therapy (IST) remains the mainstay of treatment. However, the optimal combination of available IST regimens in low-resource environments remains uncertain. Therefore, we performed a meta-analysis to evaluate the effectiveness and safety of various immunosuppressive strategies—particularly equine antithymocyte globulin (eATG) combined with cyclosporine A (CSA), with or without eltrombopag (EPAG), as well as CSA and anabolic steroids-based monotherapies, for the treatment of AA in Indian patients. Fifty-nine studies (49 single-arm, 10 multi-arm) from PubMed, Google Scholar, and Indian Society of Haematology & Blood Transfusion (ISHBT) conference proceedings from December 1995 to December 2024 were included. The primary outcomes comprised overall response rates (ORRs) at 3, 6, and 12 months. Secondary outcomes included 5-year overall survival (OS), event-free survival (EFS), and treatment-related adverse events (TRAEs). Pooled estimates were calculated using a random-effects model, and heterogeneity was assessed with I² statistics. Meta-regression was used to explore sources of variability, and sensitivity analyses assessed the robustness of the pooled outcomes. Response rates (RR) varied significantly across treatment modalities and time points. In single-arm analysis, monotherapy with CSA or anabolic steroids showed limited efficacy, with pooled ORRs at 3 months of 26.9% and 19.77%, respectively. In contrast, dual therapy with eATG plus CSA substantially improved outcomes, achieving ORRs of 45.4% at 3 months and 62.99% at 6 months. A comparable trend was observed with THYMOGAM (an Indigenous eATG) plus CSA, with ORRs of 44.7% and 59.6% at the same intervals. The most pronounced benefit was noted with triple therapy regimens. The combination of eATG + CSA + EPAG yielded the highest ORRs—63.1% at 3 months, 80.7% at 6 months, and sustained 79.4% at 12 months. THYMOGAM-based triple therapy also performed well, maintaining an ORR of approximately 52% at 3 months. At 12 months, most combination therapies showed stable or slightly reduced responses, whereas monotherapy results remained poor or unchanged. A comparative analysis of double-arm studies further revealed differences in treatment efficacy. The RRs between THYMOGAM + CSA and ATGAM + CSA at 3, 6, and 12 months were not statistically significant (OR 0.69, *p* = 0.13; OR 0.72, *p* = 0.12, and OR 0.67, *p* = 0.23, respectively). Similarly, comparative analysis of dual therapy (eATG + CSA) with triple therapy (eATG + CSA + EPAG) at 6 months showed no significant difference in ORRs (OR 0.67, *p* = 0.50). In survival analysis, the eATG + CSA and THYMOGAM + CSA demonstrated 5-year OS rates of 74.90% and 73%, respectively. The 5-year EFS rate for eATG + CSA was 63.5%. Common TRAEs included febrile neutropenia and serum sickness. Mortality rates showed no significant differences among different treatments regimens: eATG + CSA vs. ATGAM + CSA (11.31% vs. 6.24%, *p* = 0.15), THYMOGAM + CSA vs. ATGAM + CSA (13.15% vs. 6.24%, *p* = 0.17), and eATG + CSA vs. eATG + CSA + anabolic steroids (11.31% vs. 15.03%, *p* = 0.41). In conclusion, IST remains an effective frontline approach for AA patients in India, especially where transplant options are limited. Regimens incorporating EPAG offer superior response rates, and THYMOGAM presents a cost-effective yet clinically viable alternative to ATGAM. These insights support more tailored, accessible treatment strategies in resource-constrained settings.

**Supplementary information:**

The online version contains supplementary material available at 10.1007/s00277-026-06779-7.

## Introduction

AA is a rare, life-threatening hematological disorder characterized by pancytopenia with hypocellular bone marrow [1–3]. The worldwide prevalence of AA is estimated to be between 1.4 and 14 cases per million. The annual incidence among the Asian population is 2 to 3 times higher than that of the Western population, which is estimated to be 1.5 to 2.3 per million. Among the Indian population, the incidence is believed to be approximately 6.8 per million, with higher rates observed among younger individuals aged 15 to 25 and those over 60, with no discernible variation across genders or ethnicities [4–8]. Based on the Camitta criteria, AA is classified into severe aplastic anaemia (SAA), very severe aplastic anaemia (VSAA), and non-severe aplastic anaemia (NSAA). VSAA is defined by neutrophil counts < 0.2 × 10^9^/L, while SAA entails neutrophil counts < 0.5 × 10^9^/L for SAA [9–11]. NSAA can be further categorized into transfusion-dependent non-severe aplastic anaemia (TD-NSAA) and non-transfusion-dependent non-severe aplastic anaemia (NTD-NSAA), depending on the need for transfusion of blood products [12]. 

According to the International Agranulocytosis and Aplastic Anemia study (IAAS), the diagnosis of AA requires the presence of at least two of the following peripheral blood findings: (i) hemoglobin < 100 g/L, (ii) platelet < 50 × 10^9^/L, (iii) total leucocyte count < 3.5 × 10^9^/L or absolute neutrophil count < 1.5 × 10^9^/L [13, 14]. The recommended standard first-line treatment for newly diagnosed AA patients (aged < 40 years) is allogeneic BMT from an HLA-identical sibling donor and IST with a combination of Antithymocyte Globulin and CSA in patients with no HLA-matched donor and age > 40 years. Both BMT and IST can potentially restore hematopoiesis, but only BMT offers curative potential [1, 13, 14]. If IST fails in patients with an HLA-identical sibling donor who did not use transplantation as a first-line treatment, BMT remains an option as a second-line therapy [11]. In contrast, if there is no response or relapse after the first course of IST, a second course of rabbit ATG and CSA is recommended. However, it should not be administered earlier than 6 months after the initial course, as a response typically occurs within 3 to 6 months [14]. While BMT remains the sole curative treatment, this method is limited by several factors, including high costs, limited resources and expertise, and the scarcity of matched donors [13, 15, 16]. 

In the last few decades, the survival of AA has significantly improved in Western countries due to the availability of better supportive care and definitive management options for children and adults. However, in developing countries like India, managing AA remains a real-time challenge due to delays in diagnosis, a lack of proper supportive care, limited availability of expertise centres, and patients’ affordability issues [11]. ATGAM (Pfizer, USA) and THYMOGAM (Bharat Serums and Vaccines Ltd., India) are the two equine ATGs (eATG) used to treat AA. The RR of ATGAM ranges from 50 to 85% and THYMOGAM 30–70% in the Indian population. However, a study conducted in 2019 in the Eastern part of India found a similar RR of THYMOGAM and ATGAM in the adult Indian population [17]. Recently, the addition of thrombopoietin receptor agonists, such as Eltrombopag (EPAG), to the IST has improved efficacy, with an overall response rate of 80 to 87% [18–20]. 

However, the choice of therapy in resource-constrained environments often depends on availability and affordability. Many patients are treated with alternative regimens, including CSA and synthetic androgens (stanozolol, oxymetholone, and danazol). In various studies conducted in India, CSA has shown an overall response of 19.6–56%, with minimal adverse effects, making it a treatment option for patients with SAA [21–24]. On the other hand, studies on synthetic androgens have shown RR ranging from 14 to 20% only [4, 25, 26]. However, the combination therapy of androgens and CSA did not show any statistically significant response or survival benefit when compared with single-agent CSA therapy in the Indian population [27]. 

Despite established treatment guidelines, significant gaps persist in understanding the comparative efficacy and safety of these therapies in resource-constrained settings. In the context of India, these gaps are further widened by the recent unavailability of ATGAM. Currently, Thymogam, which has been available in India for the last two decades and is also accessible in some other countries, including Bolivia, Cuba, Ecuador, Guatemala, Myanmar, the Philippines, Sri Lanka, Vietnam, Pakistan, Kenya, Tanzania, Rwanda, and Mauritius, is the only available option for anti-thymocyte globulin therapy. However, no randomized controlled studies have specifically evaluated Thymogam, highlighting the urgent need for a meta-analysis to assess its efficacy and safety profile in these settings. Therefore, this systematic review and meta-analysis aim to identify the most effective and feasible treatment regimens that can aid in decision-making to meet the needs of patients in developing countries. We reviewed published and unpublished articles and conducted a systematic review and meta-analysis to evaluate and synthesize the available evidence regarding eATG, including THYMOGAM, combined with CSA (as dual therapy) or CSA + EPAG (as triple therapy) in treating AA, particularly within the Indian context. We also examined the clinical outcomes of monotherapies such as CSA and anabolic steroid drugs.

## Methods

### Literature search

#### Search strategy

Two reviewers (VC, SC) independently performed a systematic literature search in PubMed and Google Scholar from December 1995 to December 2024, following PRISMA (Preferred Reporting Items for Systematic Reviews and Meta-Analyses) guidelines. In addition, abstracts presented at the ISHBT annual conference from 2009 to 2023 were included. The search terms used for all electronic databases: *(Aplastic Anemia) AND (Aplastic Anaemia) AND (Indigenous OR Equine OR Horse AND ATG OR Thymoglobulin OR THYMOGAM OR ATGAM OR (Antithymocyte globulin)) AND (survival OR progress OR progression OR relapse OR response OR outcome OR cohort OR randomized OR comparative OR retrospective OR prospective OR trial).* This study was registered with PROSPERO (CRD420251176541).

#### Inclusion/exclusion criteria

The inclusion and exclusion criteria were articulated using the PICO (Population, Intervention, Comparison, Outcome) framework as follows:

**Population (P)**:

- Patients diagnosed with AA, including SAA, VSAA, and NSAA subtypes, were included. Both adult and pediatric patients, regardless of sex, were eligible.
- The primary focus was on the Indian population, although two additional studies from Cuba [28] and Myanmar [29] using THYMOGAM were included because of their direct relevance to indigenous equine ATG use.

**Intervention (I)**:

Eligible interventions included immunosuppressive or pharmacological regimens for AA management, specifically:

- Equine antithymocyte globulin (eATG) [ATGAM or THYMOGAM] combined with CSA, with or without adjuncts such as EPAG, romiplostim, methylprednisolone, or anabolic steroids (stanozolol, danazol).
- Monotherapies using CSA, EPAG, or anabolic steroids.

**Comparison (C)**:

- Between-regimen comparisons (THYMOGAM + CSA vs. ATGAM + CSA; eATG + CSA vs. eATG + CSA + EPAG; eATG + CSA vs. CSA).
- Single-arm studies without comparators were also included if they reported the predefined outcomes.

**Outcome (O)**:

Primary Outcome:

- ORR at 3, 6, and 12 months, defined as the proportion of patients achieving either a complete response (CR) or partial response (PR) according to study-specific hematologic criteria.

Secondary Outcomes:

- OS: Time from therapy start to death from any cause, reported as a 5-year survival rate.
- EFS: Time from therapy start to relapse, treatment failure, or death.
- TRAEs: Frequency and severity of adverse events linked to therapy, graded according to CTCAE criteria.

**Inclusion Criteria**:

- Observational, retrospective, prospective, or interventional studies evaluating pharmacologic therapy for AA with the above interventions.
- Studies reporting at least one of the defined outcomes (ORR, OS, EFS, or TRAEs).
- Publications from December 1995 to December 2024.

**Exclusion criteria**:

- Studies involving hematopoietic stem cell transplantation (HSCT) as part of therapy.
- Systematic reviews, editorials, commentaries, letters, or non-clinical (in vitro or animal) studies.
- Studies lack sufficient outcome data or do not specify treatment regimens.

### Data extraction

Two authors independently extracted relevant data from the included studies, following the predefined inclusion and exclusion criteria. 59 studies were included, comprising 49 single-arm studies and 10 double-arm studies. The studies were further categorized into subgroups based on treatment regimens. These subgroups included eATG plus CSA (eATG is further sub-grouped into THYMOGAM plus CSA or ATGAM plus CSA), eATG plus CSA and EPAG, THYMOGAM plus CSA and EPAG, eATG plus CSA combined with anabolic steroids like stanozolol or danazol, CSA alone or in combination with danazol or stanozolol, or EPAG and methylprednisolone as monotherapy. For each study, we extracted the following information: title, first author’s name, year of publication, Journal, type of study, trial duration, number of patients, baseline characteristics of patients, treatment regimens, ORR, OS, EFS, and AEs data.

### Risk of bias assessment

Risk of bias (ROB) assessment was performed for the 10 double-arm studies using the Newcastle-Ottawa Quality Assessment Scale (NOS) for cohort studies. The scale evaluates three domains: selection, comparability, and outcome. For each domain, responses were rated as **“low risk**,**” “high risk**,**” or “unclear”** based on the level of detail and methodological rigor reported in the study. ROB analysis was not performed for single-arm studies, as they lacked comparator groups and sufficient methodological detail, thereby limiting the applicability of standard ROB assessment tools.

### Statistical analysis

Statistical analysis was conducted to robustly address the study objectives and accommodate the heterogeneity inherent in studies from diverse clinical settings. Stata version 18.5 MP-Parallel Edition (StataCorp LLC, College Station, TX, USA) was used for data analysis. A random-effects model, with response rate as the effect size, was used to calculate the pooled results. OS was calculated, and survival curves were generated using a parametric survival model based on the exponential distribution. Heterogeneity was evaluated considering treatment regimen and sample size, using Chi-square tests and quantified by I² statistics. I² values of 0–40%, 30–60%, 50–90%, and 75–100% indicated low, moderate, substantial, and considerable heterogeneity, respectively. Publication bias was evaluated by Egger’s regression test and visualized through funnel plots. Meta-regression analysis explored the effects of covariates such as study design, sample size, and follow-up duration on heterogeneity. To assess the stability of pooled estimates, a sensitivity analysis (leave one out approach) was done by sequentially omitting individual studies.

## Results

Results of systematic literature search

**Fig. 1:**
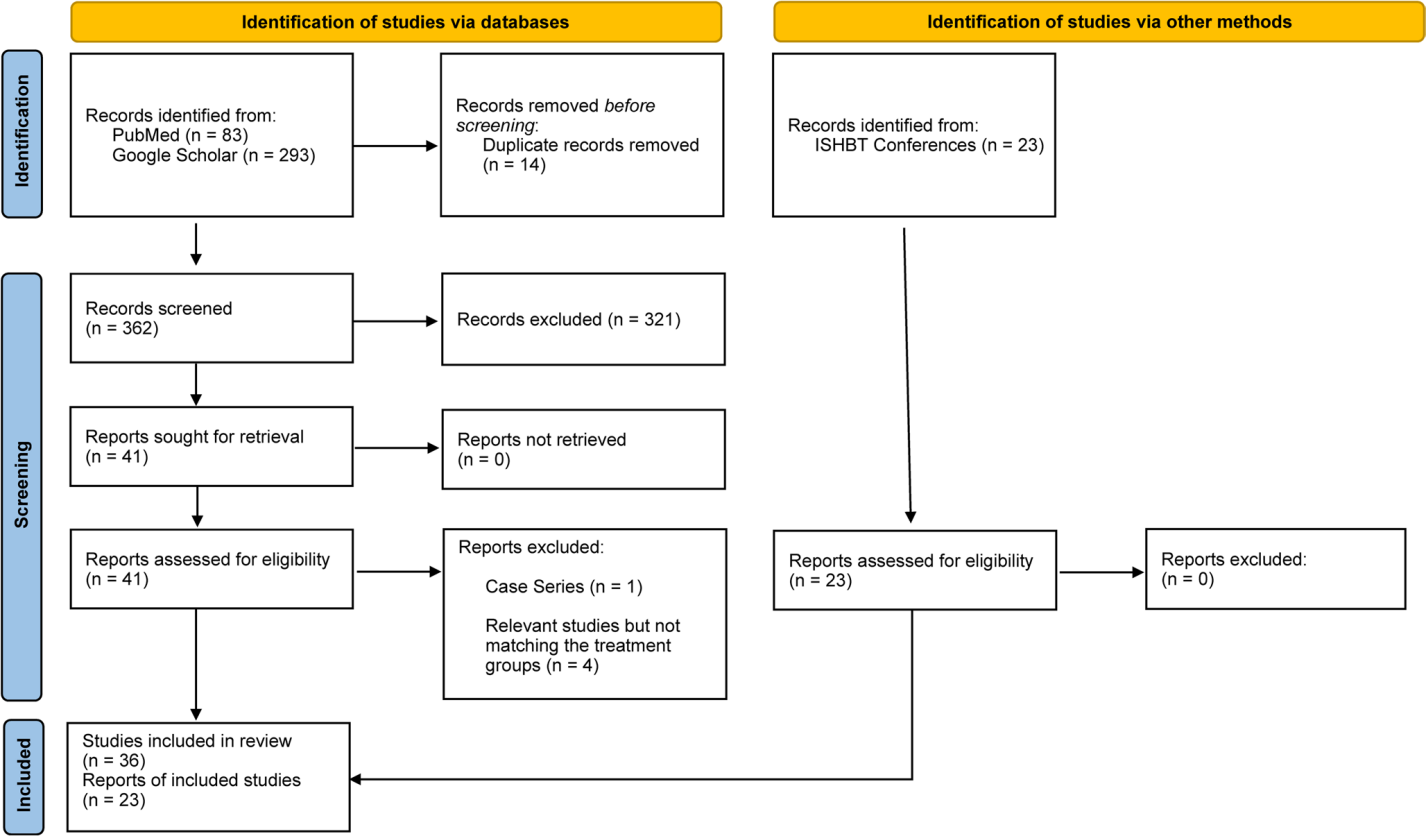
PRISMA flow diagram of the systematic literature search and its selection process

Figure 1 displays a flowchart of the search strategy and study selection. A total of 399 records were initially identified: 83 from PubMed, 293 from Google Scholar, and 23 published abstracts in the ISHBT (2009–2023). After removing 14 duplicates, 385 studies underwent title and abstract screening, and 321 were excluded as not meeting the inclusion criteria. Fifty-nine potentially relevant articles (49 single-arm and 10 double-arm studies) were eligible for meta-analysis. The characteristics of the included studies are presented in Supplementary Table 1 (ST1).

### Primary outcome findings

**Single Arm Studies**

The pooled ORRs for all treatment regimens used to treat AA were calculated at 3, 6, and 12 months. A detailed breakdown of the complete response (CR), partial response (PR), and ORRs is provided in the supplementary table **(ST2**,** SF1).**

#### Overall response rates at 3 months

**Fig. 2:**
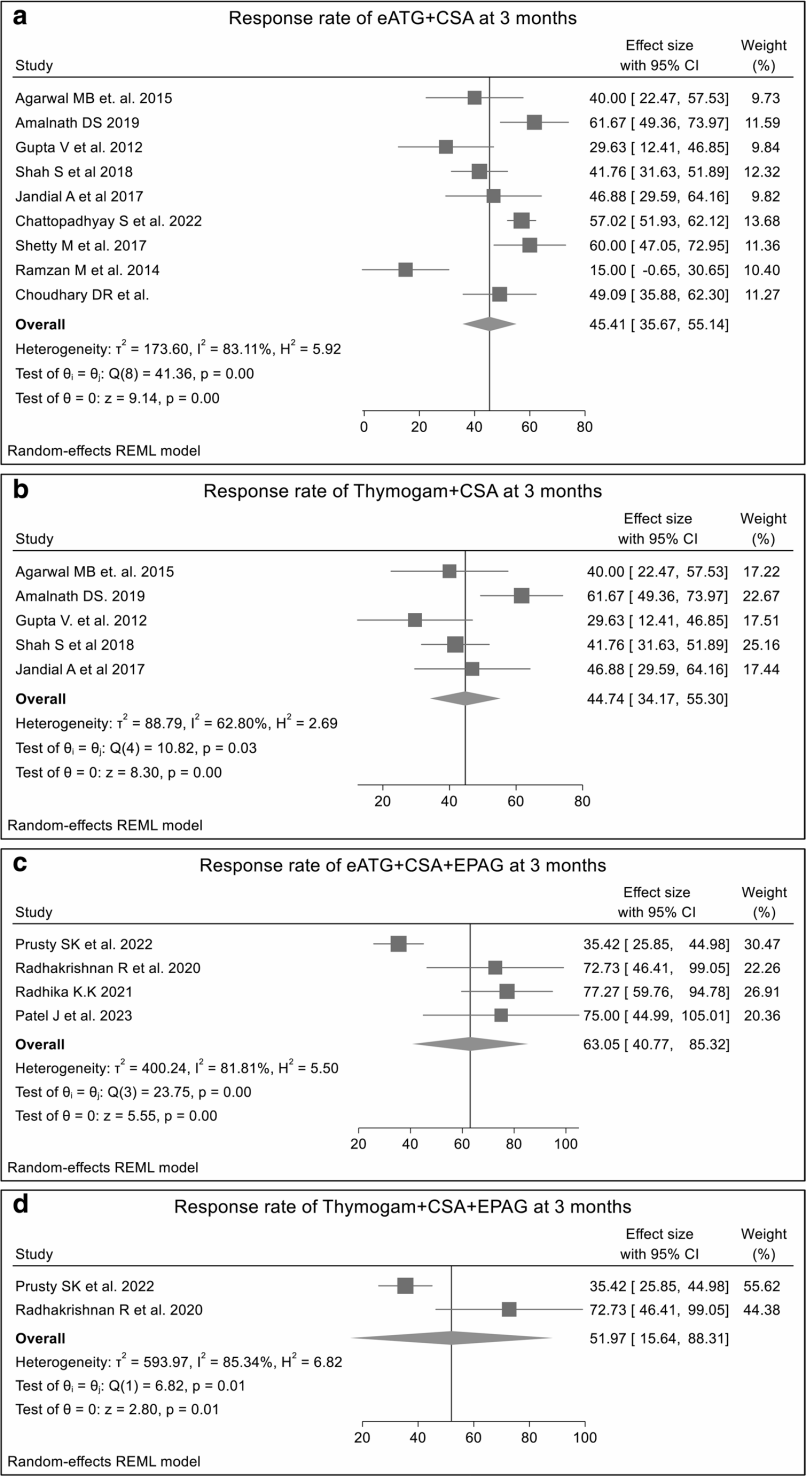
The forest plots shows ORR at 3 months for various treatment regimens across single-arm studies. Fig2A. ORR for eATG + CSA; Fig2B. ORR for THYMOGAM + CSA; Fig2C. ORR for eATG + CSA + EPAG; Fig2D. ORR for THYMOGAM + CSA + EPAG. The presence of heterogeneity across most treatment regimens underscore the influence of factors such as study design, patient population, or treatment protocols, which affect the consistency of the resultsistered eATG combined with CSA

Outcomes of monotherapy were generally lower than those of combination regimens. The pooled ORR for CSA monotherapy was 26.90% **(SF2)** and 19.77% for the anabolic steroids group **(SF3).** Dual regimens, including standard IST (eATG plus CSA) and THYMOGAM plus CSA, were associated with an improved response, yielding a pooled ORR of 45.41% and 44.74% (Fig. 2A and B**)**, respectively. The combination of eATG + CSA + anabolic steroids yielded a higher pooled ORR of 56.03% **(SF4).** The addition of EPAG to standard IST further augmented RR. A triple regimen with eATG + CSA + EPAG achieved the highest responses, with an ORR of 63.05% (Fig. 2C**).** Similarly, the THYMOGAM + CSA + EPAG regimen demonstrated an ORR of 51.97% (Fig. 2D**).**

#### Overall response rates at 6 months

At the 6-month follow-up, the RR analysis revealed higher hematologic recovery compared to the 3-month outcomes, with notable differences between monotherapies, dual therapy, and triple combination regimens. Monotherapy with CSA yielded a pooled ORR of 44.38%, *p* = 0.25. **(SF2)**, while anabolic steroids monotherapy remained largely ineffective, with a pooled ORR of only 17.25%, *p* = 0.73. **(SF3)** A study conducted by **Agarwal B.R. et al. 1995** [30] reported an ORR of 14.28% following treatment with methylprednisolone.

**Fig. 3:**
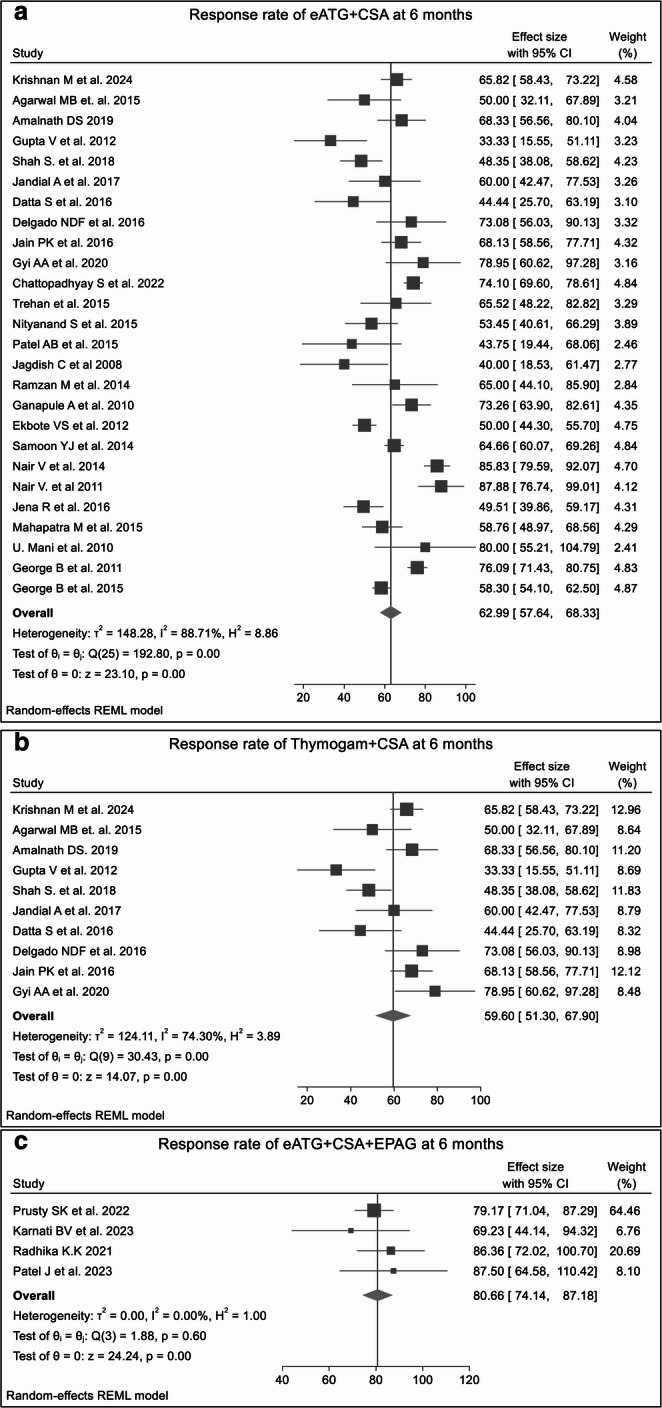
The forest plots shows ORRs at 6 months for various treatment regimens across single-arm studies. Fig3A. ORR for eATG + CSA; Fig3B. ORR for THYMOGAM + CSA; Fig3C. ORR for eATG + CSA + EPAG. Combination therapies, particularly eATG + CSA + EPAG, demonstrated higher ORR than CSA or steroid-only treatments. The presence of heterogeneity across most treatment regimens underscore the influence of factors such as study design, patient population, or treatment protocols, which affect the consistency of the results

Among dual therapies, the combination of eATG + CSA demonstrated a significant improvement in ORR (62.99%, *p* < 0.0001), showing a favorable increase relative to the response at 3 months (Fig. 3A, **SF5).** The CSA + anabolic steroids therapy exhibited an ORR of 55.71% **(SF6)**. Notably, ATGAM + CSA (68.61%) **(SF6)** therapy responded better than THYMOGAM + CSA (59.60%) (Fig. 3B**)**, although the difference did not reach statistical significance (*p* = 0.22). **(SF8)** Triple-drug combination therapies further enhanced hematologic recovery beyond that seen at 3 months. The combination of eATG + CSA + EPAG achieved the highest pooled ORR of 80.66% (*p* = 0.14) (Fig. 3C, **SF9)** followed by eATG + CSA + anabolic steroids (ORR 65.71%, *p* = 0.33) **(SF4)** and eATG + CSA + Romiplostim (ORR 66.57%). **(SF10).**

#### Overall response rates at 12 months

**Fig. 4:**
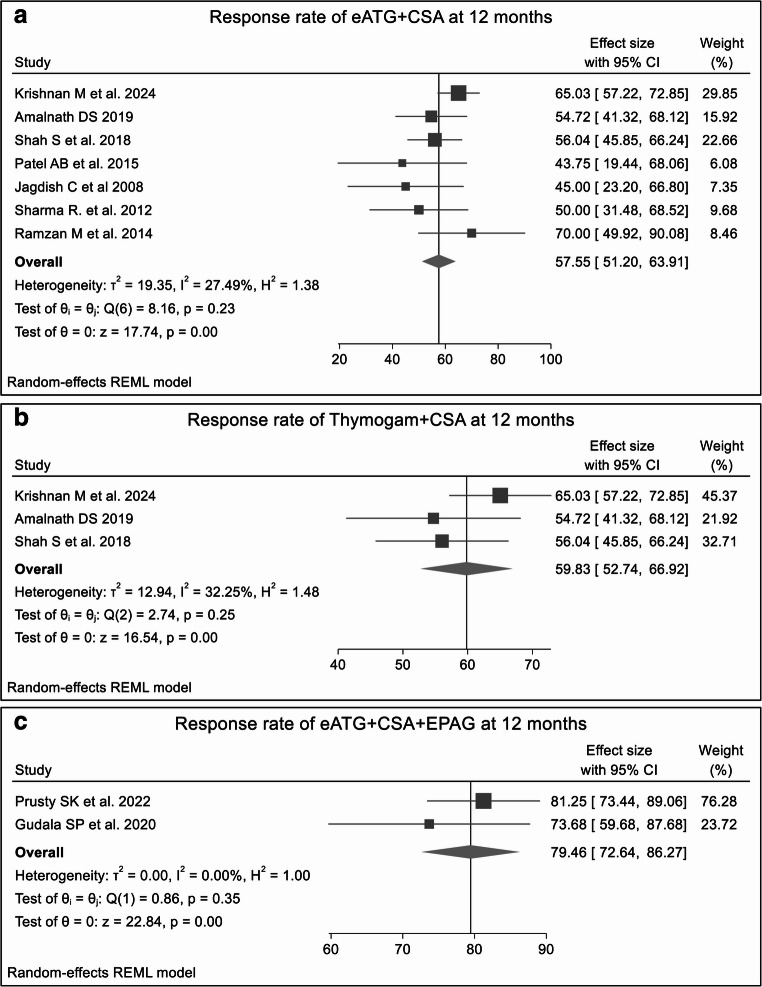
The forest plots shows ORRs at 6 months for various treatment regimens across double studies. Fig4A. ORR for eATG + CSA; Fig4B. ORR for Thymogam + CSA; Fig4C. ORR for eATG + CSA + EPAG. Combination therapies, particularly eATG + CSA + EPAG, demonstrated higher ORR than other treatment regimens. The presence of heterogeneity across most treatment regimens highlights the influence of factors such as study design, patient population, or treatment protocols, affecting the consistency of the results

Compared to the overall response at 6 months, anabolic steroids monotherapy showed no significant improvement at 12 months (ORR 17.25% vs. 20%, *p* = 0.79). **(SF11)** The combination of eATG + CSA demonstrated a slight decrease in ORR relative to the 6-month response (62.99 vs. 57.55%, *p* = 0.20). **(**Fig. 4A, **SF12)** Similarly, the THYMOGAM plus CSA subgroup exhibited a negligible change in ORR from 59.83% at 12 months to 59.60% at 6 months, *p* = 0.97.**(**Fig. 4C, **SF13)** In contrast, the triple-drug regimen of eATG + CSA + EPAG maintained a consistent ORR over time (79.40% at 12 months vs. 80.66% at 6 months, *p* = 0.72). **(**Fig. 4C, **SF14)**

##### Double-arm studies

**Fig. 5:**
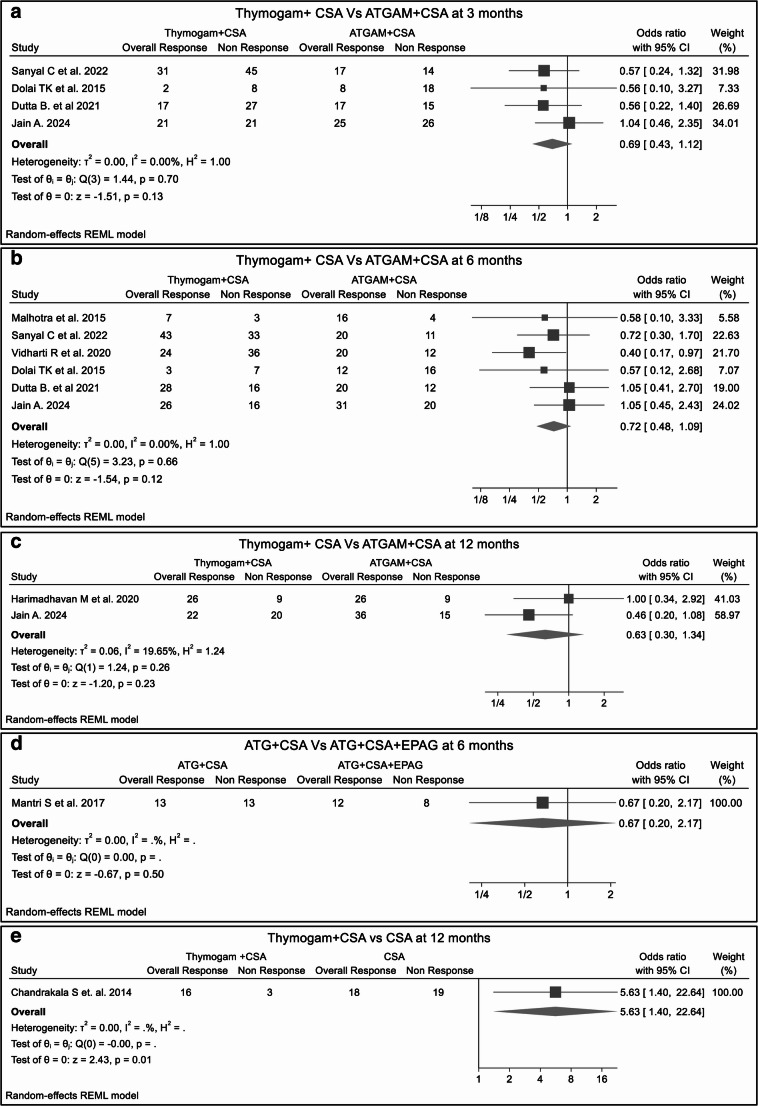
The forest plots presents 3 and 6-month ORRs comparing double-arm treatment regimens across double studies. Risk ratios generally favor combination therapies, though no significant differences are observed between groups. Fig5A. ORR between THYMOGAM+CSA vs ATGAM+CSA at 3 months; Fig5B. ORR between THYMOGAM+CSA vs ATGAM+CSA at 6 months; Fig5C. ORR between THYMOGAM+CSA vs. ATGAM+CSA at 12 months; Fig5D. ORR between eATG + CSA vs. eATG+CSA+EPAG at 6 months; Fig5E. ORR between THYMOGAM+CSA vs. CSA at 12 months; Despite potential variability in study design, patient characteristics, or treatment protocols, low heterogeneity across comparisons suggests consistent findings across studies

In a comparative analysis of double-arm studies, the RR at 3, 6, and 12 months between two treatment groups did not reach statistical significance; patients treated with THYMOGAM + CSA were 31% less likely to achieve a response than those treated with ATGAM + CSA (RR 0.69, *p* = 0.13) after 3 months (Fig. 5A**)**, 28% (RR 0.72, *p* = 0.12) after 6 months (Fig. 5B**)** and 37% (RR 0.63, *p* = 0.23) after 12 months (Fig. 5C**)**. Similar results were obtained comparing the response of dual therapy (eATG + CSA) versus triple therapy (eATG + CSA + EPAG) at 6 months (RR = 0.67, *p* = 0.50). (Fig. 5D**)** A single study comparing eATG + CSA versus CSA group showed no significant superiority in response rate (RR = 1.30, *p* = 0.77) at 3 months **(SF15);** however, another study comparing THYMOGAM + CSA with CSA monotherapy patients showed a significantly superior response (RR 5.73, *p* = 0.01) after 12 months of treatment. (Fig. 5E**).**

### Secondary outcome findings

Overall survival and Event-free survival

**Fig. 6:**
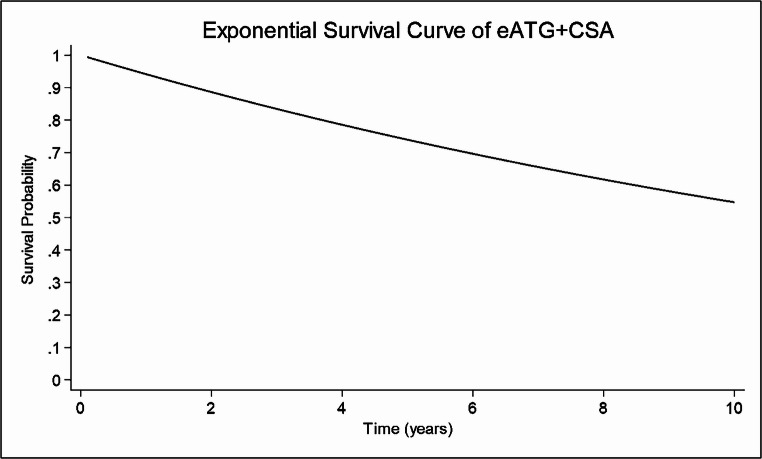
Five years exponential survival curve for patients administered eATG combined with CSA

The exponential survival curves for the four treatment groups show different patterns in long-term survival outcomes under the assumption of a constant hazard over time, which suggests stable risk profiles throughout the follow-up period. Patients treated with ATGAM + CSA showed a 5-year survival rate of 78.22% **(SF16)**, followed closely by those receiving eATG + CSA and THYMOGAM + CSA, with survival rates of 74.90% **(**Fig. 6**)** and 73%, respectively. **(SF17)** However, the difference in OS rate among the three groups is not statistically significant (eATG + CSA vs. ATGAM + CSA, *p* = 0.59, (**SF18);** ATGAM + CSA vs. THYMOGAM + CSA, *p* = 0.62, **SF19**), suggesting similar therapeutic outcomes between ATGAM and both THYMOGAM and eATG regimens. Conversely, the eATG + CSA + steroids group showed a markedly lower survival probability, with only 40% surviving at 3 years. **(SF20)** Two studies analyzed the 5-year EFS of eATG + CSA 63.48%. **(SF21)**

### Publication bias evaluation – Funnel plots

**Fig. 7:**
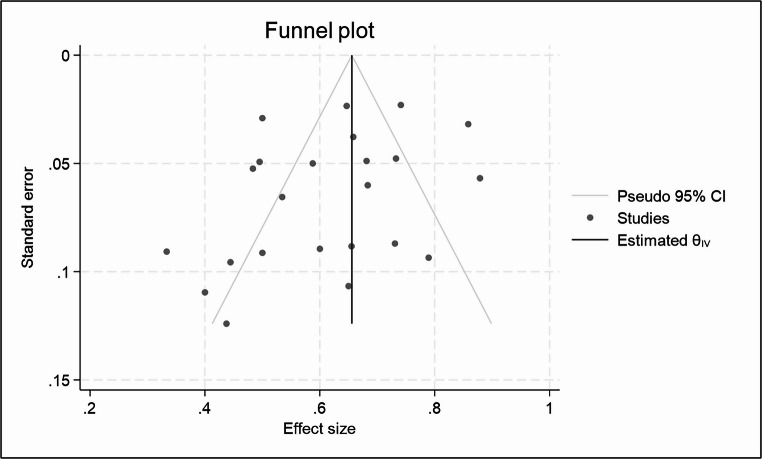
Funnel chart showing the overall response rate between patients treated with eATG combined with CSA

The p-values (0.0622 and 0.4515) were greater than 0.05, indicating no strong statistical evidence of publication bias based on Egger’s test. (Fig. 7**and SF22**)

### Risk of bias assessment

**Fig. 8:**
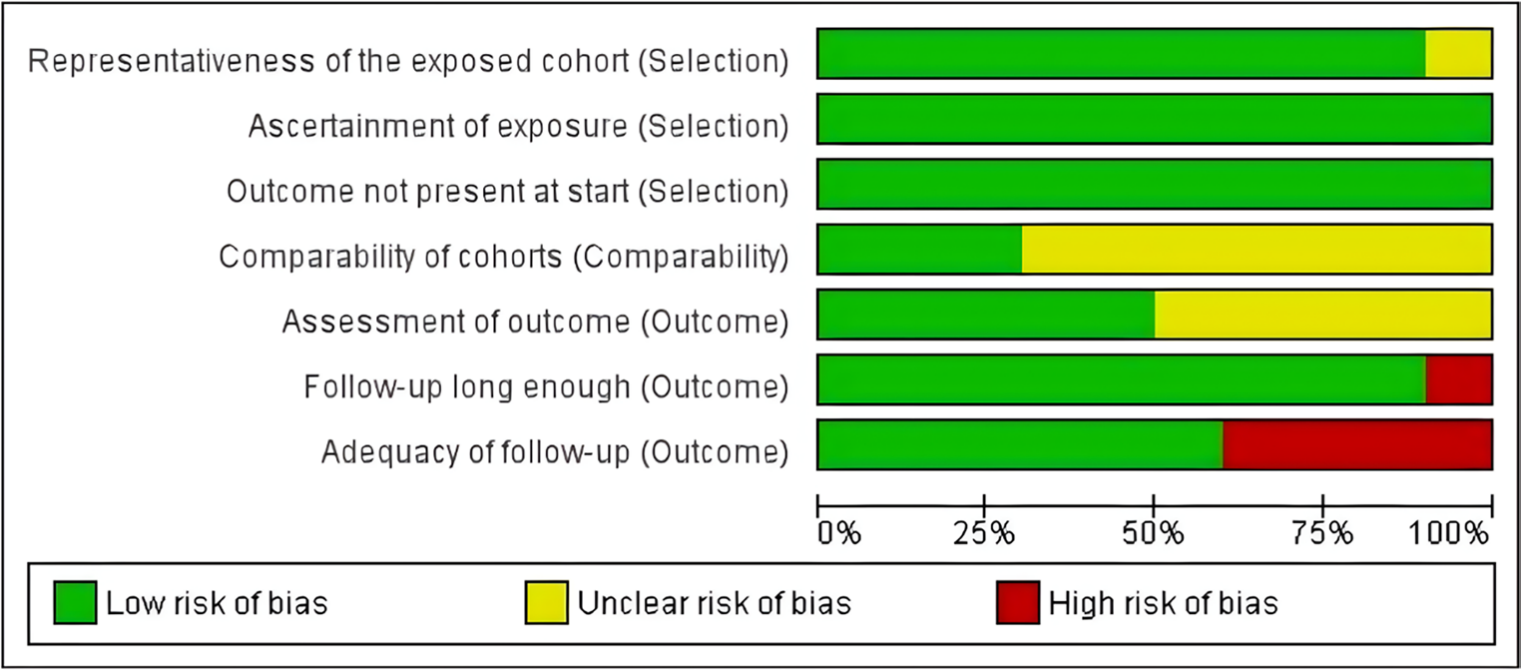
The bias risk evaluation results of the included double-arm studies

ROB was performed using Review Manager 5.3 software. The analysis revealed that due to limited reporting—particularly in 7 studies available only as conference abstracts—several criteria could not be fully assessed and thus were marked as “unclear” (Fig. 8). Most domains show a low risk of bias (green), while follow-up-related aspects, such as “Adequacy of follow-up” and “Follow-up long enough,” display higher proportions of unclear (yellow) and high risk (red) of bias. (**ST4)**

### Subgroup analysis

**Fig. 9:**
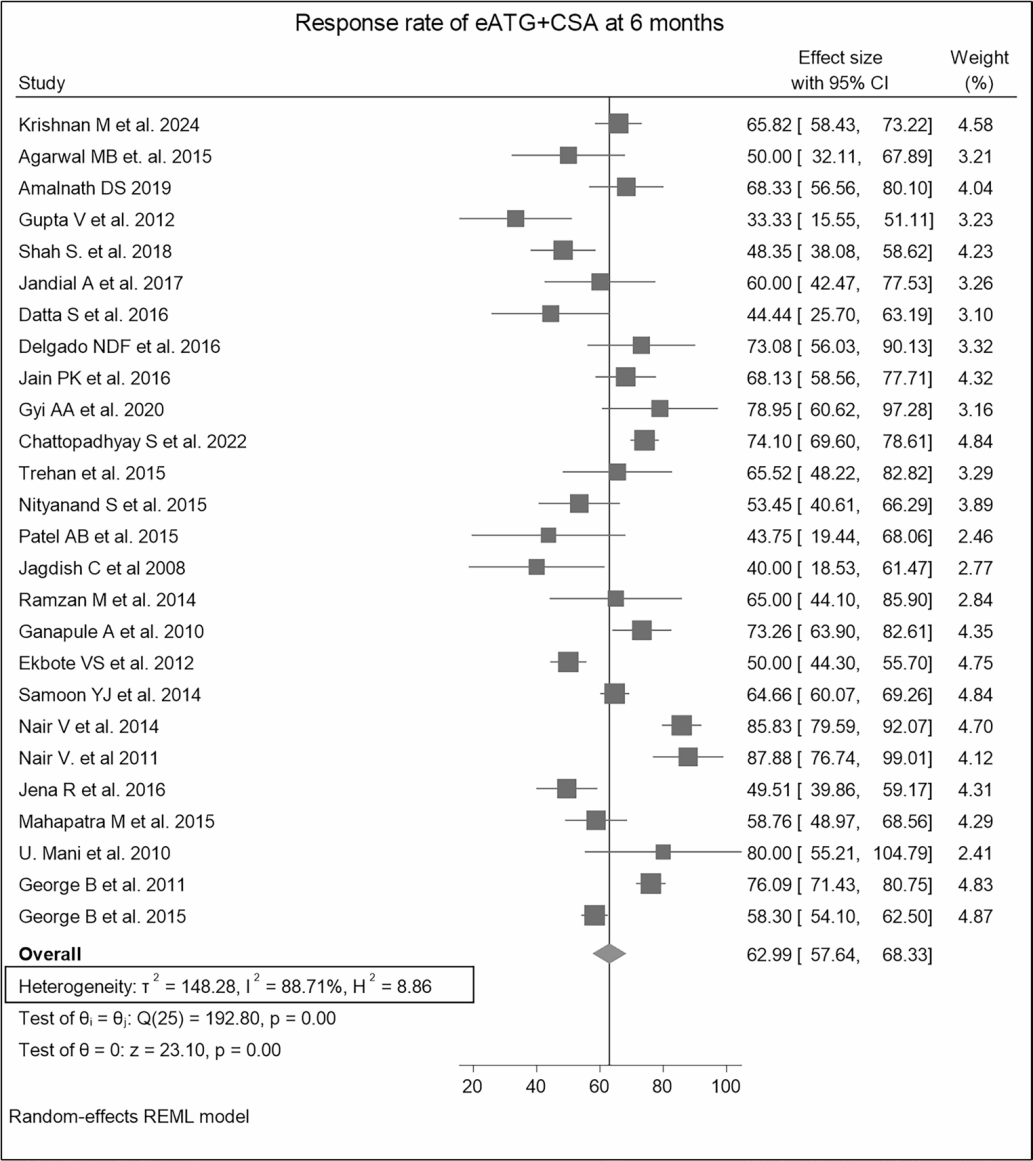
Heterogeneity Analysis for eATG + CSA at 6 Months

**Fig. 10:**
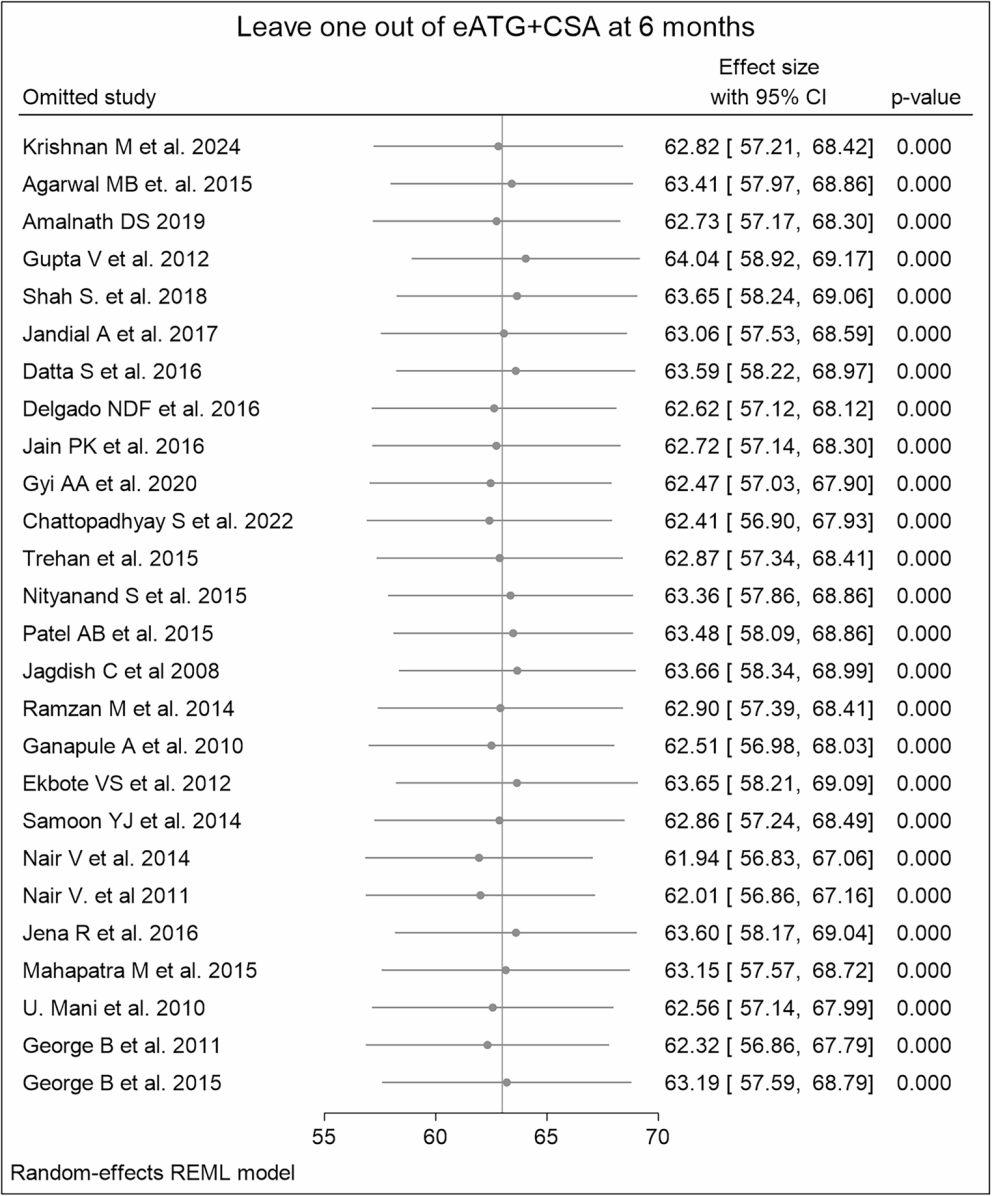
Leave-One-Out Sensitivity Analysis for eATG + CSA at 6 Months

Q statistics and I^2^ were used to test the heterogeneity and revealed high variability among the studies in some treatment groups. The pooled heterogeneity was 88.71% for eATG + CSA (Fig. 9), 74.30% for THYMOGAM + CSA, 94.65% for ATGAM + CSA, and 93.45% for the CSA-only group at 6 months, with all p-values < 0.0001. **(ST3)** Leave-one-out sensitivity analyses for treatment groups demonstrated robust and consistent pooled estimates in most regimes. (Fig. 10) However, for the CSA-only group at 3 months, the exclusion of certain studies resulted in wider confidence intervals and non-significant p-values in some cases, suggesting a relatively less stable estimate for this subgroup. Double-arm analyses showed no substantial changes in pooled risk ratios upon study omission, maintaining overall statistical robustness. **(S4-S7)**.

Additionally, meta-regression analysis was performed using variables such as study design, sample sizeand follow-up to identify potential sources of heterogeneity; however, it did not reveal any statistically significant predictors contributing to heterogeneity **(S8)**.

### Safety

A safety analysis was conducted for single-arm studies for all the various treatment regimens in AA, and adverse events (AEs) were categorized based on severity using the Common Terminology Criteria for Adverse Events (CTCAE). In the eATG + CSA group, the most frequently observed TRAEs were serum sickness (9.52%), gum hypertrophy (3.22%), acute kidney injury (2.96%), and febrile neutropenia (2.70%). The THYMOGAM + CSA group reported febrile neutropenia (9.48%), gum hypertrophy (8.75%), serum sickness (4.41%), and hypertension (3.28%) as the most prevalent AEs. In contrast, the ATGAM + CSA group noted serum sickness (11.11%) and arthralgia (5%) as the most common AE. The mortality rate was 11.70% for eATG + CSA + EPAG, followed by THYMOGAM + CSA (13.15%), eATG + CSA (11.31%), and ATGAM + CSA (6.24%). (Table 1) However, the difference in mortality rates was not statistically significant. (Table 2). In double-arm studies, safety analysis was not conducted due to the unavailability of AE data or lack of stratified reporting between comparison groups. Only one study reported the mortality rate: 31.57% for THYMOGAM + CSA and 21.62% for CSA [42]. A detailed breakdown of adverse events by different treatment regimens is provided in the supplementary Table **(ST4).**

**Table 1:** Most common *Treatment-Related* adverse events reported in different treatment regimens

S. No.	Intervention	No. of studies	Total no. of patients	Most common TRAEs – *n* (%)
1	eATG + CSA	24	1922	Serum Sickness – 183 (9.52) Gum Hypertrophy – 62 (3.22) Acute Kidney Injury – 57 (2.96) Febrile Neutropenia – 52 (2.70) Death – 225 (11.31)
2	THYMOGAM + CSA	9	548	Febrile Neutropenia – 52 (9.48) Gum Hypertrophy – 48 (8.75) Serum sickness – 24 (4.41) Hypertension – 18 (3.28) Death – 76 (13.15)
3	ATGAM + CSA	4	360	Serum sickness – 40 (11.11) Arthralgia – 18 (5) Lower respiratory tract infection – 10 (2.77) Death – 25 (6.24)
4	IST (eATG + CSA) + EPAG	6	188	Hyperbilirubinemia – 21 (11.17) Transaminitis – 12 (6.38) Death – 27 (11.70)
5	THYMOGAM + CSA + EPAG	2	107	Hyperbilirubinemia – 7 (6.54) Transaminitis – 8 (7.47) Death – 19 (17.75)
5	IST (eATG + CSA) + Anabolic Steroids	3	519	Jaundice – 23 (4.43) Death – 12 (15.03)
6	CSA Only	3	92	Gum Hypertrophy – 9 (9.78) Renal Dysfunction – 6 (6.52) Death – 3 (3.26)
7	Anabolic steroids	2	74	Death – 15 (20.27)
8	IST (eATG + CSA) + Romiplostim	1	12	Febrile Neutropenia – 3 (25) Death – 2 (16.66)

**Table 2:** Mortality rates comparison between different treatment regimens

S. No.	Interventions	Mortality Rates (%)	*p*-values
1.	eATG + CSA vs. THYMOGAM + CSA	11.31 vs. 13.15	0.69
2.	eATG + CSA vs. ATGAM + CSA	11.31 vs. 6.24	0.15
3.	ATGAM + CSA vs. THYMOGAM + CSA	6.24 vs. 13.15	0.17
4.	eATG + CSA vs. eATG + CSA + EPAG	11.31 vs. 11.70	0.93
5.	eATG + CSA vs. eATG + CSA + anabolic steroids	11.31 vs. 15.03	0.41

## Discussion

This comprehensive meta-analysis, encompassing 59 studies and over 6000 patients from diverse regions in India, provides a robust evaluation of IST in AA within a resource-limited setting. The analysis covered a wide range of regimens, including standard dual therapy (eATG plus CSA), newer triple therapy combinations featuring thrombopoietin receptor agonists (TPO-RAs) like eltrombopag (EPAG), and alternative monotherapy strategies.

Our findings highlight that monotherapy, particularly CSA alone or anabolic steroids, resulted in limited clinical effectiveness, with a pooled 3-month ORR of 26.90% for CSA and 19.77% for anabolic steroids. Although CSA showed some improvement by 6 months (44.38%), the group using anabolic steroids showed no improvement. These results underscore its limited efficacy compared to combination regimens. This aligns with prior reports from Indian and international cohorts indicating suboptimal long-term outcomes with CSA monotherapy alone [21–24]. 

Conversely, standard treatment with IST showed a better ORR of 45.41% at 3 months and 62.01% at 6 months; similar outcomes were seen with THYMOGAM + CSA, which had ORRs of 44.74% and 59.60% at 3 and 6 months, respectively. These findings reaffirm the critical role of ATG-based immunosuppression as the current standard of care for moderate to severe AA, consistent with global treatment guidelines [1, 11, 13]. Importantly, adding EPAG to the standard IST further augmented hematologic recovery. Our pooled analysis revealed that the triple combination of eATG with CSA and EPAG achieved the highest response rate, with ORRs of 63.05% at 3 months and 80.66% at 6 months, significantly higher than dual therapy alone. These findings are consistent with multiple recent global meta-analyses, which have reported that IST combined with EPAG results in significantly higher ORR and complete response (CR) rates than IST alone [6, 10, 12]. For example, **Illango et al.** demonstrated an ORR of 86% with IST plus EPAG compared to 74% with IST alone [6]. Similarly, **Zhang et al.** reported a pooled odds ratio (OR) of 2.10, *p* < 0.00001 at 3 months and a pooled OR of 2.13, *p* < 0.008 at 6 months, favoring IST + EPAG over IST alone [10]. In another meta-analysis, patients treated with ATG combined with EPAG showed significant improvement in 6-month OR (OR = 3.66, 95% CI 2.39–5.61, *P* < 0.001) compared to those administered with IST alone [12]. In our analysis, this superiority persisted at 12 months, where eATG + CSA + EPAG maintained an ORR of 79.40%, supporting the long-term hematologic benefits of EPAG addition.

Comparative analysis of ATGAM versus THYMOGAM combined with CSA suggested a trend toward higher RR with ATGAM (RR = 0.693, *p* = 0.13 at 3 months, although statistical significance was not achieved. This observation is consistent with previous studies; however, patient factors, healthcare infrastructure, and the availability of supportive care may attenuate real-world differences in outcomes. For instance, the cost of ATGAM is up to twice that of the indigenous THYMOGAM, making the latter a more accessible option in resource-limited settings [13]. Importantly, these findings suggest that THYMOGAM remains a viable alternative to ATGAM in settings where its cost and availability are limiting factors.

In long-term survival analysis, ATGAM plus CSA demonstrated a 5-year OS rate of 78.22%, followed by eATG plus CSA (74.90%), and THYMOGAM and CSA (73%). Although these differences were not statistically significant, the findings suggest that none of the treatment regimens is inferior to the others. These results support previous studies suggesting that IST, particularly regimens based on eATG, yields lasting responses and prolonged survival for patients with AA who are ineligible for hematopoietic stem cell transplantation (HSCT) [31, 32].

The ROB assessment highlighted unclear risks in several domains, particularly outcome ascertainment and follow-up adequacy. However, the overall impact of these limitations appears modest, as corroborated by sensitivity analyses and meta-regression. The leave-one-out sensitivity analyses showed consistent effect sizes across most treatment subgroups. However, greater variability was observed in the CSA-only group, reflecting its inherent clinical heterogeneity and the relatively fewer high-quality studies. In double-arm comparisons, risk ratios remained stable on omission analyses, with no single study materially altering the pooled effects. Further, meta-regression analyses did not identify significant covariates contributing to between-study heterogeneity. These findings reinforce the overall robustness of the meta-analysis while underscoring the need for cautious interpretation in subgroups with less consistent outcomes.

Despite being broadly efficacious, IST regimens carried distinct adverse event (AE) profiles. Febrile neutropenia was the most common AE, particularly among THYMOGAM-treated patients (9.48%) and serum sickness in the ATGAM cohort (11.11%). Interestingly, mortality rates varied across regimens, with ATGAM + CSA demonstrating a mortality rate of 6.24%, while THYMOGAM + CSA showed a mortality rate of 13.15% (*p* = 0.17). However, this difference was not statistically significant, indicating that both treatment regimens offer comparable outcomes.

These findings carry significant clinical implications. In resource-limited settings where BMT is unattainable for many patients, IST—especially with EPAG—provides a very effective alternative. The improved ORRs observed with IST + EPAG therapy indicate its potential for better outcomes in AA patients in India and similar settings. Given the unavailability of ATGAM, THYMOGAM presents the most viable alternative, striking a balance between effectiveness and cost, although further direct comparative trials are needed.

### Limitations

Despite rigorous methodology and merit considerations, several limitations exist. Substantial heterogeneity was observed across included studies, stemming from differences in diagnostic criteria, patient demographics, treatment protocols, and supportive care practices. The publication bias was insignificant, and the predominance of observational studies and conference abstracts may have introduced reporting biases. Publication bias and meta-regression analysis were only performed for treatment groups with more than 10 studies. Additionally, in meta-regression analysis age was excluded as a covariate due to inconsistent reporting across studies—age categories overlapped, varied in range definitions, or lacked summary values—preventing reliable standardization and risking biased or misleading results. Severity was also excluded, as most studies included mixed-severity populations without separate analyses by severity level. Moreover, the lack of standardized reporting of adverse events across studies precludes a comprehensive comparative safety analysis.

## Conclusion

In conclusion, this meta-analysis provides robust evidence supporting the efficacy of combination immunosuppressive regimens in the treatment of AA within the Indian clinical context. The analysis revealed that eATG-based IST with cyclosporine A (CSA) significantly outperformed monotherapies in terms of hematologic response at 3, 6, and 12 months. Specifically, eATG + CSA and THYMOGAM + CSA achieved pooled ORRs of approximately 45–62% by 6 months, establishing them as effective and accessible first-line regimens in settings where hematopoietic stem cell transplantation is not feasible.

Notably, adding EPAG to these dual regimens resulted in substantially higher overall ORRs. The triple-drug therapy of eATG (including THYMOGAM) + CSA + EPAG demonstrated the most pronounced efficacy, with a pooled ORR of 80.66% at 6 months and 79.40% at 12 months. This suggests that the combination therapy (eATG + CSA + EPAG) administered to AA patients yields a higher hematologic response and overall survival, decreases patient dependence on blood products, shortens the average hospital stay, reduces the economic burden on patients, and thus improves patient quality of life. THYMOGAM represents a cost-effective and clinically comparable alternative to ATGAM, broadening therapeutic access. Our findings provide valuable insights into treatment options for AA, supporting the continued use of IST/IST + EPAG in transplant-ineligible patients and underscoring the need for further research to improve treatment methods and long-term outcomes.

## Supplementary Information

Below is the link to the electronic supplementary material.

Supplementary Material 1 (DOCX 88.9 KB)

Supplementary Material 1 (DOCX 850 KB)

## Data Availability

No datasets were generated or analysed during the current study.

## Abbreviations

AA: Aplastic Anaemia
AE: Adverse Event
ALG: Antilymphocyte Globulin
ATG: Antithymocyte Globulin
BMT: Bone Marrow Transplantation
BSH: British Society for Haematology
CR: Complete Response
CSA: Cyclosporine A
CTCAE: Common Terminology Criteria for Adverse Events
eATG: Equine Antithymocyte Globulin
EFS: Event-Free Survival
EPAG: Eltrombopag
HLA: Human Leukocyte Antigen
HSCT: Hematopoietic Stem Cell Transplantation
IAAS: International Agranulocytosis and Aplastic Anemia Study
IAP: Indian Academy of Pediatrics
ISHBT: Indian Society of Haematology & Blood Transfusion
IST: Immunosuppressive Therapy
NOS: Newcastle-Ottawa Scale
NSAA: Non-Severe Aplastic Anaemia
NTD-NSAA: Non-Transfusion-Dependent NSAA
OR: Odds Ratio
ORR: Overall Response Rate
OS: Overall Survival
PRISMA: Preferred Reporting Items for Systematic Reviews and Meta-Analyses
ROB: Risk of Bias
RR: Relative Risk
SAA: Severe Aplastic Anaemia
ST: Supplementary Table
TPO-RA: Thrombopoietin Receptor Agonist
TD-NSAA: Transfusion-Dependent NSAA
VSAA: Very Severe Aplastic Anaemia
