# Supplementary material for: A meta-analysis of immunosuppressive and Pharmacological therapies in aplastic anaemia with and without Indigenous equine antithymocyte globulin (eATG)
Source: Ann Hematol. 2026 Jan 22;105(2):52. doi: 10.1007/s00277-026-06779-7 (PMC12827372; doi:10.1007/s00277-026-06779-7)
Supplement: Supplementary file 1 — Supplementary Material 1 (DOCX 88.9 KB) [file 277_2026_6779_MOESM1_ESM.docx]

**Table ST1:** The general characteristics of the included studies:

| **S. No.** | **Author** | **Study Design** | **Duration of the study** | **Total no. of patients** | **Age (years)** | **Intervention** | **Dose** |
| --- | --- | --- | --- | --- | --- | --- | --- |
| 1 | Jain et al. 2024 (1) | Retrospective | 2003-2021 | 93 | > 12 | Low/High dose eATG + CSA | eATG 25/40 mg/kg/day  CSA 5 mg/kg/day |
| 2 | Kotha A et al. 2010 (2) | Prospective | - | 31 | - | eATG + CSA  CSA alone | - |
| 3 | Gupta D et al. 2022 (3) | Retrospective | 2015-2021 | 72 | - | eATG+CSA  eATG + CSA + EPAG  CSA  CSA + EPAG | - |
| 4 | Chattopadhyay S et al. 2022 (4) | Retrospective | 1989-2020 | 363 | 16 - 74 | eATG + CSA | - |
| 5 | Ekbote VS et al. 2012 (5) | Retrospective | 1985-2012 | 296 | 3- 83 | eATG + CSA | - |
| 6 | Ganapule A et al. 2010 (6) | Retrospective | 1985-2009 | 86 | 15 - 83 | eATG + CSA | - |
| 7 | Krishnan M et al. 2025 (7) | prospective | 2016-2022 | 158 | > 13 | Thymogam+CSA | Thymogam 25 mg/kg/day  CSA 5 mg/kg/day |
| 8 | Agarwal MB et al. 2014 (8) | Phase IV, prospective, multicenter | 2011-2013 | 30 | > 2 | Thymogam+CSA | Thymogam 40mg/kg/day  CSA 4-6 mg/kg/day |
| 9 | Amalnath DS 2019 (9) | Retrospective | 2016-2018 | 60 | 13 - 63 | Thymogam+CSA | Thymogam 40mg/kg/day CSA 5 mg/kg/day |
| 10 | Gupta V et al. 2012 (10) | Retrospective | 5 year | 27 | 4 - 14 | Thymogam+CSA | Thymogam 15mg/kg/day  CSA 5 mg/kg/day |
| 11 | Shah S et al. 2018 (11) | Retrospective case records | 2012-2015 | 91 | 2 - 67 | Thymogam+CSA | Thymogam 20/40 mg/kg/day  CSA 10 mg/kg/day |
| 12 | Jandial A et al. 2017 (12) | Retrospective | 2010-2016 | 34 | 13-45 | eATG + CSA | eATG 25 mg/kg/d for 4 days  CSA 40 mg/kg/d for 4 days |
| 13 | Datta S et al. 2016 (13) | Prospective | 2015-2016 | 27 | 12 - 60 | Thymogam+CSA | Thymogam 40mg/kg/day  CSA 5 mg/kg/day |
| 14 | Chandrakala S. et al. 2014 (14) | Retrospective | 2008-2014 | 85 | 24 (median age) | Thymogam + CSA  CSA | Thymogam 40 mg/kg/day for 4 days |
| 15 | Delgado NDF, 2016 (15) | Observational | 2000-2012 | 26 | 18 - 74 | Thymogam+CSA | Thymogam 20 mg/kg/day  CSA 5 mg/kg/day |
| 16 | Jain PK et al. 2016 (16) | Retrospective | 2012-2015 | 91 | Median age  Adults - 31; Children - 9 | Thymogam+CSA | Thymogam 40mg/kg/day  CSA 5 mg/kg/day |
| 17 | Trehan A et al. 2015 (17) | Retrospective | 2008-2014 | 29 | 1.5 - 13 | eATG + CSA | eATG 40mg/kg/day CSA |
| 18 | Nityanand S. et al. 2015 (18) | Prospective | 2012-2014 | 58 | - | eATG + CSA | - |
| 19 | Samoon YJ et al. 2014 (19) | Retrospective | 1986-2013 | 416 | 16-73 | ALG + CSA or ATGAM + CSA | - |
| 20 | Nair V. et al 2013 (20) | Retrospective | 1996-2009 | 120 | > 18 | ATGAM+CSA | ATGAM 40mg/kg/day CSA 8-10 mg/kg/day |
| 21 | Nair V. et al 2011 (21) | Prospective | 1998-2009 | 33 | <18 years | eATG + CSA | eATG 40mg/kg/day CSA 8-10 mg/kg/day |
| 22 | Mahapatra M et al. 2015 (22) | Retrospective | 2007-2014 | 1501 | 2 - 83 | eATG + CSA | eATG 40mg/kg/day CSA 5mg/kg/day |
|  | Mahapatra M et al. 2015 | - | 2007-2010 | 97 | 27 | ATGAM | ATGAM 40 mg/kg/day |
|  | Mahapatra M et al. 2015 | - | - | 31 | - | ATG (THYMOGAM) | Thymogam 40 mg/kg/day |
|  | Mahapatra M et al. 2015 | - | - | 59 |  | CSA + Stanozolol | CSA 5 mg/kg/day Stanozolol 2 mg/kg/day |
| 23 | Jena R et al. 2016 (23) | Retrospective | 2012-2013 | 104 | 7 -63 | eATG + CSA | eATG 40 mg/kg/day  CSA 6 mg/kg/day |
| 24 | Dutta B. et al. 2021 (24) | Retrospective and prospective observational | 2013-2018 | Total 76  Thymogam 44  ATGAM 32 | 6-66 | Thymogam + CSA  ATGAM + CSA | CSA 5 mg/kg/day |
| 25 | Malhotra P et al. 2015 (25) | Retrospective | 2003-2010 | Total 39  Thymogam 16  ATGAM 23 | 13–67 | Thymogam+CSA  ATGAM + CSA | Thymogam 40 mg/kg/day × 4 days ATGM 25 mg/kg/day × 4 days CSA 5 mg/kg/day |
| 26 | Ramzan M et al. 2014 (26) | Retrospective | 2005-2012 | 20 | <18 | Thymogam+CSA  ATGAM + CSA | Thymogam/ATGM 40 mg/kg/day × 5 days CSA 8-10 mg/kg/day |
| 27 | Sanyal C. et al. 2022 (27) | Prospective | 2011-2022 | Total 107  Thymogam 71.1%  ATGAM 28.9% | 8-66 yrs | Thymogam/ATGAM + CSA | eATG 40 mg/kg/day × 4 days  CSA 5 mg/kg/day |
| 28 | Harimadhavan M et al 2020 (28) | Retroprospective | 2015-2020 | Total 70  Thymogam 35  ATGAM 35 | 0-60 years | Thymogam/ATGAM + CSA | - |
| 29 | Vidharti R et al 2020 (29) | Prospective | 2011-2020 | Total 92  Thymogam 60  ATGAM 32 | 8–66 yrs | Thymogam/ATGAM + CSA | eATG 40 mg/kg/day × 4 days  CSA 5 mg/kg |
| 30 | Dolai TK et al. 2015 (30) | Prospective | 2011-2015 | Total 36  Thymogam 10  ATGAM 26 | 6-65 yrs | Thymogam/ATGAM + CSA | Thymogam/ATGAM 40 mg/kg/day  CSA 5 mg/kg |
| 31 | Sharma R. et al. 2012 (31) | Retrospective | 2001-2009 | Total 35  Thymogam 24  ATGAM 5  Lymphoglobulin 6 | 5-12 yrs | Lymphoglobulin / ATGAM / Thymogam + CSA | ATGAM and Lymphoglobulin 15 mg/kg for 5 days  Thymogam 40 mg/kg for 5 days  CSA 10 mg/kg/day |
| 32 | Jagdish C et al 2008 (32) | Prospective | 2001-2005 | 23 | 6 -12 | Lymphoglobulin / ATGAM / Thymogam + CSA | eATG 15 mg/Kg/day for 5 days CSA 10 mg/Kg/day orally |
| 33 | Choudhary DR et al. (33) | Retrospective | 2003-2005 | Total 61  Thymogam 11  ATGM 48  ALG 2 | 8-75 yrs | ATGAM / ALG / Thymogam + CSA | eATG 15 mg/Kg/day for 5 days CSA 3-5 mg/Kg/day orally |
| 34 | Prusty SK et al. 2022 (34) | Prospective | - | 96 | >50 years | eATG + CSA + EPAG | - |
| 35 | Radhakrishnan R et al. 2020 (35) | Prospective observational | - | 11 | 56 | Thymogam + CSA + ETP | Thymogam 40 mg/kg/day  EPAG 150 mg once daily  CSA 5 mg/kg/day |
| 36 | Radhika K.K 2021 (36) | Retrospective | 2019-2020 | 22 | 7-65 yrs | eATG + CSA + ETP | eATG (15 patients) 40 mg/Kg/day for 5 days  eATG (7 patients) 15 mg/Kg/day for 5 days  CSA 5 mg/Kg/day |
| 37 | Patel J et al. 2023 (37) | Prospective | 2020-2022 | - | - | eATG + CSA + EPAG | eATG 40 mg/Kg/day for 4 days  EPAG 150 mg/day  CSA 5 mg/Kg/day |
| 38 | Gudala SP et al. 2020 (38) | Retrospective | 2019-2020 | 38 | 7-68 yrs | eATG + CSA + EPAG | EPAG 75/150 mg/day |
| 39 | Karnati BV et al. 2023 (39) | Retrospective Observational | - | 13 | 17-70 yrs | eATG + CSA + EPAG | eATG 15mg/kg |
| 40 | Kumar R et al. 2004 (40) | Retrospective | 2001-2003 | 440 | 19 | Stanozolol / CSA / ATG / Combination | ATG 75–80mg/kg over 5-8 days  Stanozolol 2 mg/kg/d CSA 3mg/kg/d |
| 41 | Dhingra G et al. 2023 (41) | Retrospective | 2021-2022 | 12 | 8-65 yrs | eATG + CSA | eATG 40 mg/Kg/day for 4 days  CSA 5 mg/Kg/day |
| 42 | Balasubramanian R et al. 2023 (42) | Retrospective | 2010-2023 | 44 | 3-77 yrs | Thymogam ATG + CSA + Danazol | - |
| 43 | Kumar N et al. 2024 (43) | Prospective observational | 2018-2019 | 35 | 10.77 ± 3.6 | eATG, Steroids, and CSA | ATG 40 mg/Kg/day for 4 days  CSA 5 mg/Kg/day |
| 44 | U. Mani et al. 2010 (44) | Prospective | - | 10 | - | eATG + Methylprednisolone + CSA | eATG 40 mg/Kg/day for 5 days Methyl Prednisolone 250 mg/day |
| 45 | Korula A et al. 2015 (45) | Retrospective | 1991-2014 | 379 | 2-77 yrs | CSA | - |
| 46 | Shetty M et al. 2016 (46) | Prospective | - | 20 | 27 | CSA | 5 mg/kg/day |
| 47 | Varma S et al. 1999 (47) | 1998-1999 | Prospective | 15 | 8-34 yrs | CSA | 5-7.5 mg/ kg |
| 48 | Mandal PK et al. 2017 (48) | Prospective | 2012-2014 | 57 | 6-81 yrs | CSA | 5 mg/kg/day |
| 49 | M Rai et al. 2001 (49) | Prospective | - | 12 |  | CSA + Stanozolol | CSA 6 mg/kg/day  Stanozolol 1 mg/kg/day |
| 50 | Kamat G et al. 2022 (50) | Retrospective | 2017-2020 | 26 | 11-80 yrs | CSA + Danazol / EPAG | CSA 50–100 mg  Danazol 100–200mg  EPAG 150 mg |
| 51 | Datta A et al. 2010 (51) | Retrospective | 2008-2009 | 25 | Age >12 years | Danazol | Danazol 600mg/day |
| 52 | Marwaha RK et al. 2004 (52) | Retrospective case-record analysis | 1991-2000 | 49 | 9 Years | Stanozolol | 1 mg/kg/day |
| 53 | Agarwal BR et al. 1995 (53) | Retrospective | - | 7 | 3-11 years | High Dose Methyl Prednisolone (HDMP) | 30 mg/kg/day for 4 days,  20 mg/kg/day for 3 days,  10 mg/kg/day for 7 days,  5 mg/kg/day for 7 days and 2.5 mg/kg/day for 7 days |
| 54 | Patel AB et al. 2015 (54) | Retrospective | 2011-2012 | 18 | 7-58 yrs | eATG+CSA | eATG 40 mg/kg for 8 days & 80 mg/kg for 4 days  CSA 10 mg/kg |
| 55 | Shetty M et al. 2017 (55) | Prospective | 2011-2016 | 55 | 29 mean age | eATG+CSA | eATG 15mg/Kg/day for 5 days or 40mg/kg/day for 4 days CSA 5mg/Kg/day orally |
| 56 | Mantri S et al. 2017 (56) | Retrospective | 2015-2016 | 46  eATG+CSA 26  eATG+CSA+EPAG 20 |  | eATG+CSA //  eATG+CSA+EPAG | - |
| 57 | Gyi AA et al. 2020 (57) | Retrospective | 2018-2020 | 19 | 14-57 yrs | Thymogam+CSA // EPAG | Thymogam 40-160mg/kg  (9 patients 40-60 mg/kg  5 patients 60-80 mg/kg  5 patients 160 mg/kg)  CSA 5 mg/kg/day  EPAG 100mg/day for 8 weeks |
| 58 | George B. et al 2011 (58) | Retrospective | 1985-2010 | 322 | 12 to >50 | eATG+CSA+Methylprednisolone | 40 mg/kg/day × 4 days or Lymphoglobulin (Pasteur Merieux, France) at 15 mg/kg/day × 5 days  CSA 6 mg/kg/day |
| 59 | George B. et al 2015(59) | Prospective | 1985 - 2013 | ATGAM – 278  ALG - 252 | 1.5 – 74 years | ATGAM + CSA  ALG + CSA | ATGAM – 15 mg/kg/day  ALG – 40 mg/kg/day  CSA – 3 mg/kg/day |

**References:**

1. Jain A, Jandial A, Mani T, Kishore K, Singh C, Lad D, et al. Comparable outcomes with low-dose and standard-dose horse anti-thymocyte globulin in the treatment of severe aplastic anemia. Blood Res. 2024 Dec;59(1):6.

2. Kotha A, Narendra AMVR, Prasad K, Mallikarjuna S, Srinivasan VR. Managing Aplastic Anemia in India: ATG Plus Cyclosporin Versus Cyclosporin Alone. Indian J Hematol Blood Transfus. 2010 Dec;26(4):165–6.

3. Gupta D, Moule P, Agarwal C, Sivaprakasam Y, Balasubramanian R, Verma M, et al. Clinical Profile and Treatment Outcome of Severe Aplastic Anemia in Adults: An Experience from Tertiary Care Centre in North India. Indian J Hematol Blood Transfus. 2022 Nov;38(S1):75–75.

4. Chattopadhyay S, Lionel S, Sclvarajan S, Devasia AJ, Kulkarni U, Fouzia N, et al. Anti-Thymocyte Globulin-Based Immunosuppressive Therapy in Acquired Aplastic Anemia: Long Term Outcomes. Indian J Hematol Blood Transfus. 2022 Nov;38(S1):77.

5. Ekbote VS, George B, Mathews V, Abraham A, Ahmed R, Kavitha M, et al. Antilymphocyte (ALG)/Antithymocyte Globulin (ATG) for Severe Aplastic Anemia—Single Centre Experience. Indian J Hematol Blood Transfus. 2012 Dec;28(4):207.

6. Ganapule A, George B, Mathews V, Abraham A, Ahmed R, Kavitha M, et al. Immunosuppresive Therapy with Antilymphocyte (ALG)/ Antithymocyte Globulin (ATG) for Aplastic Anemia: A Single Centre Experience. Indian J Hematol Blood Transfus. 2010 Dec;26(4):177.

7. Krishnan M, Amalnath D. Response to Immunosuppressive Therapy in Aplastic Anemia—A Single Centre, Prospective Study of 158 Patients from a Tertiary Care Centre in India. Indian J Hematol Blood Transfus [Internet]. 2024 Jun 17 [cited 2024 Nov 8]; Available from: https://doi.org/10.1007/s12288-024-01794-y

8. Agarwal MB, Jijina F, Shah S, Malhotra P, Damodar S, Ross C. Safety and Efficacy of Indigenous Equine Antithymocyte Globulin Along with Cyclosporine in Subjects with Acquired Aplastic Anemia. Indian J Hematol Blood Transfus. 2015 Jun;31(2):174–9.

9. Amalnath DS. Response to Horse ATG (Thymogam, Bharat Serums and Vaccine, India) and Cyclosporine in Aplastic Anemia: A Single Centre, Retrospective Study of 60 Patients from Southern India. Indian J Hematol Blood Transfus. 2020 Jul;36(3):473–6.

10. Gupta V, Kumar A, Tilak V, Saini I, Bhatia B. Immunosuppressive Therapy in Aplastic Anemia. Indian J Pediatr. 2012 Dec;79(12):1587–91.

11. Shah S, Jain P, Shah K, Patel K, Parikh S, Patel A, et al. Immunosuppressive therapy for aplastic anemia: a single-center experience from western India. Ann Hematol. 2019 Jan;98(1):41–6.

12. Jandial A, Malhotra P, Mishra K, Nampoothiri RV, Lad DP, Sachdeva MUS, et al. Efficacy of Low Dose Generic Antithymocyte Globulin and Cyclosporine in Idiopathic Aplastic Anemia. Blood. 2017 Dec 8;130:4955.

13. Datta S, Kalantri S, Nayak UA, Samanta S, Sankar Ray S, Bhattacharyya M. Aplastic Anemia: Response to Indigenous Anti-thymocyte Globulin—A Single Tertiary Care Centre Experience. Indian J Hematol Blood Transfus. 2016 Nov;32(S2):12.

14. Shanmukhaiah C, Jijina F, Bombarde S, Patil R. Single Centre Experience with Indigenous Equine Anti-thymocyte Globulin in Aplastic Anaemia. Indian J Hematol Blood Transfus. 2014 Nov;30(S2):448–546.

15. Fernández Delgado ND, Blanco Coto A. Immunosuppressive triple-therapy using Tymogam® in idiopathic aplastic anemia. Rev Cuba Hematol Inmunol Hemoter. 2016;32(1):86–98.

16. Jain PK, Panchal HP, Anand AS, Patel AA, Parikh SK, Prajapathi K, et al. Outcome of Acquired Aplastic Anemia Treated with Indigenous Equine Antithymocyte Globulin (Thymogam) Along with Cyclosporine at Gujarat Cancer Research Institute (GCRI): A Regional Centre Experience. Indian J Hematol Blood Transfus. 2016 Nov;32(S2):78.

17. Trehan A, Bansal D, Varma N. Acquired Aplastic Anemia: Outcome with Immunosuppressive Therapy in Children. Indian J Hematol Blood Transfus. 2015 Oct;31(S1):26.

18. Nityanand S. Outcome of Immunosuppressive Therapy in Patients with Acquired Aplastic Anemia: Experience from a Tertiary Care Hospital in North India. Indian J Hematol Blood Transfus. 2015 Oct;31(S1):26–7.

19. Samoon YJ, Viswabandya A, Abraham A, Ganapule A, Fouzia N, Korula A, et al. Response to Immunosuppressive Therapy in a Large Cohort of Adult Patients with Aplastic Anemia. Indian J Hematol Blood Transfus. 2014 Nov;30(S2):27.

20. Nair V, Sharma A, Das S, Sondhi V, Sharma S. Immunosuppressive therapy in adults with aplastic anaemia: single-institution experience from India. Postgrad Med J. 2013 Sep;89(1055):508–15.

21. Nair V, Sondhi V, Sharma A, Das S, Sharma S. Survival after immunosuppressive therapy in children with aplastic anemia. Indian Pediatr. 2012 May;49(5):371–6.

22. Mahapatra M, Singh PK, Agarwal M, Prabhu M, Mishra P, Seth T, Tyagi S, Patil HP, Saxena R. Epidemiology, Clinico-Haematological Profile and Management of Aplastic Anaemia: AIIMS Experience. J Assoc Physicians India. 2015 Mar;63(3 Suppl):30-5. PMID: 26529865.

23. Swain, Trupti. (2016). Anti Thymocyte globulin therapy for treatment of Aplastic Anemia- A retrospective Study. International Journal of Medical and Health Sciences. 5. 225-229.

24. Dutta B, Dolai TK, Mandal PK, Baul S, De R, Senthil K, et al. Response to Immunosuppressive Therapy in Acquired Aplastic Anaemia: Experience of a Tertiary Care Centre from Eastern India. Indian J Hematol Blood Transfus. 2021 Apr;37(2):197–203.

25. Malhotra P, Bodh V, Guru Murthy GS, Datta AK, Varma N, Varma S. Outcomes of immunosuppressant therapy with lower dose of antithymocyte globulin and cyclosporine in aplastic anemia. Hematology. 2015 May;20(4):239–44.

26. Ramzan M, Yadav SP, Zafar MSH, Dinand V, Sachdeva A. Outcome of Pediatric Acquired Aplastic Anemia: A Developing World Experience. Pediatr Hematol Oncol. 2014 Feb;31(1):29–38.

27. Chirasree S, Ghosh K, Roy S, Maurya A, Baneerjee A, Bhattacharya S, et al. Outcome of Immunosuppressive Therapy in Patients with Acquired Aplastic Anaemia: Experience from a Tertiary Care Hospital in West Bengal. Indian J Hematol Blood Transfus. 2022 Nov;38(S1):78.

28. Harimadhvan M, Nataraj K, Bhat S, Prabhu S, Shah A, Komaravelli SK, et al. Response to Equine Anti-thymocyte Globulin in Aplastic Anaemia: Study from a Tertiary Care Referral Centre. Indian J Hematol Blood Transfus. 2020 Nov;36(S1):99.

29. Vidhatri R, Shekhawat PS, Mitra S, Bhattacharyaa S, Baul SN, Mondal PK, et al. Outcome of Immunosuppressive Therapy in Patients with Idiopathic Aplastic Anaemia: Experience from a Tertiary Care Centre. Indian J Hematol Blood Transfus. 2020 Nov;36(S1):100.

30. Dolai TK, Chakrabarti P, Dutta S, De R, Mandal PK. Outcome of Aplastic Anemia Patients Treated With Horse Anti Thymocytic Globulin (Atg) and Cyclosporine– An Institutional Experience. Indian J Hematol Blood Transfus. 2015 Oct;31(S1):62.

31. Sharma R, Chandra J, Sharma S, Pemde H, Singh V. Antithymocyte globulin and cyclosporine in children with aplastic anemia: a developing country experience. J Pediatr Hematol Oncol. 2012 Mar;34(2):93-5. doi: 10.1097/MPH.0b013e31823c287b. PMID: 22278201.

32. Chandra J, Naithani R, Ravi R, Singh V, Narayan S, Sharma S, et al. Antithymocyte globulin and cyclosporin in children with acquired aplastic anemia. Indian J Pediatr. 2008 Mar;75(3):229–33.

33. Dharma G, Okur H, Sayl T. Response to antithymocyte globulin therapy in severe aplastic anemia: a single center study from india. Abstract 264; Poster 171.

34. Prusty SK, Biswal M, Panda JK, Jena RK. Efficacy of Triple Drugs Combination of Hatg, Cyclosporine and Eltrombopag in Primary Severe Aplastic Anemia (PSAA): Experience From Prospective Study in Single Institution. Indian J Hematol Blood Transfus. 2022 Nov;38(S1):77–8.

35. Nair, Ragesh & Singh, Pawan & Halder, Rohan & Gambhir, Isha & Randhawa, Kiran & Bhar, Vikrant & Zahier, Ahmad & Sharma, Himanshu & MK, Amalkumar & M, Raveendran & Kaur, Amandeep. (2020). INDIGENOUS EQUINE ANTITHYMOCYTE GLOBULIN, CYCLOSPORINE AND ELTROMBOPAG COMBINATION THERAPY IN THE TREATMENT OF SEVERE APLASTIC ANEMIA.

36. Radhika KK, Bacchu S. Real World Single Center Experience of Eltrombopag Added to Standard Immunosuppressive Therapy, among Drug Naive Indian Patients with Aplastic Anemia. 2021;28(04).

37. Patel J, Kheni S, Katharotiya M, Bhise S, Motwani N, Shah D, et al. Our Experience of Triple Therapy in Severe Aplastic Anemia. Indian J Hematol Blood Transfus. 2023 Nov;39(S1):17.

38. Gudala SP, Dhawan R, Aggarwal M, Kumar P, Seth T, Mahapatra M. Addition of Eltrombopag to Immunosuppressive Therapy (ATG, Cyclosporine) in Acquired Aplastic Anemia: A Tertiary Care Center Experience. Indian J Hematol Blood Transfus. 2020 Nov;36(S1):11.

39. Karnati BV, Bhuyan B, Behera PK. Outcomes of Low Dose Anti Thymocyte Globulin (ATG) Therapy in Aplastic Anemia in a Resource Poor Setting. Indian J Hematol Blood Transfus. 2023 Nov;39(S1):1–199.

40. Kumar R, Choudhary DR, Mahapatra M, Kotwal A, Mathur A, Gopal K, et al. A Retrospective Hospital Based Study of 440 Patients of Aplastic Anemia from India: Epidemiology; Response to Therapy; and Possible Significance of Jaundice during Therapy. Blood. 2004 Nov 16;104(11):5299.

41. Dhingra G, Rajoreya A. A Single-Centre Experience of First-Line Romiplostim and Immunosuppressive Therapy in Patients With Aplastic Anemia. Cureus. 2023 Apr 17;15(4):e37682. doi: 10.7759/cureus.37682. PMID: 37206485; PMCID: PMC10190113.

42. Balasubramanian R, Thiagarajan KV, Annamalai A, Veerasamy A. Treatment of Aplastic Anemia Patients with Thymogam ATG+Cyclosporine A+Danazol from a Tertiary Care Centre in South India. Indian J Hematol Blood Transfus. 2023 Nov;39(S1):34.

43. Kumar N, Hemal A, Sangwan A, Arora SK. Outcomes after Immunosuppressive Therapy for Aplastic Anemia: A Single Centre Experience from Northern India. Indian J Hematol Blood Transfus [Internet]. 2024 Oct 29 [cited 2024 Nov 30]; Available from: https://doi.org/10.1007/s12288-024-01913-9.

44. Mani U, Banerjee D, Dey NB, Biswas SK, Ahmed R, Chandra S. Treatment of Severe Hypoplastic Anemia with ATG, Cyclosporine and Methyl Prednisolone. Indian J Hematol Blood Transfus. 2010 Dec;26(4):180.

45. Korula A, Fouzia N, Srivastava A, Mathews V, George B. Cyclosporine Monotherapy For The Treatment of Aplastic Anemia. Indian J Hematol Blood Transfus. 2015 Oct;31(S1):53.

46. Shetty M, Narendra AM, Adiraju KP, Modugu NR. Study of Aplastic Anaemia with Cyclosporine in Resource Poor Setting. J Clin Diagn Res. 2016 Jun;10(6):OC15-8. doi: 10.7860/JCDR/2016/16144.7952.

47. Varma S, Varma N, Malhotra P, Singh S, Sharma DR. Cyclosporin A monotherapy in young Indian aplastic anaemia patients. J Indian Med Assoc. 1999 Dec;97(12):505-6. PMID: 10638130.

48. Mandal PK, Baul S, Dolai TK, De R, Chakrabarti P. Outcome of Cyclosporine Monotherapy in Patients of Aplastic Anemia: Experience of a Tertiary Care Hospital in Eastern India. Indian J Hematol Blood Transfus. 2017 Mar;33(1):144–7.

49. Rai M, Singh VP, Shukla J, Sundar S, Jha VC. Low dose cyclosporine-a therapy in severe aplastic anaemia. J Assoc Physicians India. 2001 Oct;49:966–9.

50. Kamat G, Renukaradhya K Math, Goni D, Balikai G, Savanur A, Mudennavar N, et al. Use of Cyclosporine A and danazol in treatment of aplastic anemia: A real-world data from a teaching hospital in South India. Asian J Med Sci. 2022 Oct 1;13(10):223–6.

51. Datta A, Malhotra P, Varma N, Suri V, Ahluwalia J, Kumari S, et al. Response to Danazol in Patients with Aplastic Anemia. Indian J Hematol Blood Transfus. 2010 Dec;26(4):169.

52. Marwaha RK, Bansal D, Trehan A, Varma N. Androgens in Childhood Acquired Aplastic Anaemia in Chandigarh, India. Trop Doct. 2004 Jul;34(3):149–52.

53. Agarwal BR, Gulvady A, Bhalla K, Dalvi R, Currimbhoy ZE. Treatment of aplastic anemia in children with high dose methyl prednisolone. Indian Pediatr. 1995 Oct;32(10):1061–5.

54. Patel A, Patel A, Parikh S, Shah S, Panchal H, Anand A. Acquired severe aplastic anemia treated with antithymocyte globulin and cyclosporine: An experience of regional cancer center, Western India. J Appl Hematol. 2015;6(2):53.

55. Shetty M, Modugu NR, Venkata Raja AM. Study of Response to Treatment with Antithymocytic Globulin (Atg) +Cyclosporine (Csa) In Aplastic Anemia. Sch. J. App. Med. Sci., Mar 2017; 5(3c):852-858.

56. Mantri S, Chandrakala S, Jijina F, Rajesh P. A Retrospective Study to Assess the Safety and Efficacy of Addition of Eltrombopag to Standard Immunosuppressive Therapy in Patients with Aplastic Anaemia. Indian J Hematol Blood Transfus. 2017 Nov;33(S1):8.

57. Gyi AA, Htut KT, Han YN, Khine MM, Thandar HM, Thant YM. Outcome of Severe Aplastic Anemia Treated with Low Dose Immunosuppression in Resource Limited World during Pandemic: Single Center Experience from Myanmar. Blood. 2020 Nov 5;136(Supplement 1):27–27.

58. George B, Mathews V, Lakshmi KM, Ahmed R, Abraham A, Srivastava A. Response to Immunosuppressive Therapy with Antithymocyte Globulin (ATG) In Older Patients with Aplastic Anemia. Blood. 2011 Nov 18;118(21):4374.

59. George B, Mathews V, Viswabandya A, Abraham A, Ganapule A, Fouzia NA, Korula A, Lakshmi KN, Chandy M, Srivastava A. Immunosuppressive Therapy and Bone Marrow Transplantation for Aplastic Anaemia--The CMC Experience. J Assoc Physicians India. 2015 Mar;63(3 Suppl):36-40. PMID: 26529866.
