# Supplementary material for: A meta-analysis of immunosuppressive and Pharmacological therapies in aplastic anaemia with and without Indigenous equine antithymocyte globulin (eATG)
Source: Ann Hematol. 2026 Jan 22;105(2):52. doi: 10.1007/s00277-026-06779-7 (PMC12827372; doi:10.1007/s00277-026-06779-7)
Supplement: Supplementary file 2 — Supplementary Material 1 (DOCX 850 KB) [file 277_2026_6779_MOESM2_ESM.docx]

**SINGLE ARM ANALYSIS**

**Overall Response Rates**

**ST2** - Summary of overall response rates (ORR) for different treatment regimens in aplastic anemia based on single-arm studies.

| **Single-Arm Analysis – Response rates** | | | | | | |
| --- | --- | --- | --- | --- | --- | --- |
| **Treatments** | | **No. of studies** (studies in which CR and PR data are separately given) | **CR (%)** | **PR (%)** | **Overall Response Rate (%)** | **p-values (Comparing ORRs from 3 to 6 months and 6 to 12 months between the same treatment group)** |
| Equine ATG (eATG) Based | eATG + CSA (3 months) | 7 | 7.91 | 35.31 | 45.41** |  |
|  | eATG + CSA (6 months) | 20 | 15.83 | 45.83 | 62.99*** | <0.0001 |
|  | eATG + CSA (12 months) | 7 | 12.57 | 44.98 | 57.55 | 0.20 |
|  | eATG + CSA+ EPAG (3 months) | 4 | 16.73 | 40.31 | 63.05 |  |
|  | eATG + CSA+ EPAG (6 months) | 4 | 38.72 | 40.78 | 80.66 | 0.14 |
|  | eATG + CSA + EPAG (12 months) | 2 | 30.96 | 45.76 | 79.46 | 0.80 |
|  | eATG + CSA + anabolic steroids (3 months) | 2 | 30.85 | 24.74 | 56.03 |  |
|  | eATG + CSA + anabolic steroids (6 months) | 1 | 11.43 | 54.29 | 65.71 | 0.33 |
|  | eATG + CSA + Romiplostim (6 months) | 1 | 25 | 41.57 | 66.57 |  |
| CSA only | CSA (3 months) | 4 | 8.47 | 11.99 | 26.90 |  |
|  | CSA (6 months) | 5 | 10.84 | 30.38 | 44.38 | 0.25 |
| CSA + anabolic steroids | CSA + anabolic steroids (6 months) | 1 | 8.47 | 37.29 | 55.71* |  |
| Thymogam Based | Thymogam+CSA (3 months) | 4 | 1.67 | 39.45 | 44.74* |  |
|  | Thymogam+CSA (6 months) | 8 | 13.39 | 42.28 | 59.60** | <0.0001 |
|  | Thymogam+CSA (12 months) | 3 | 12.97 | 46.86 | 59.83 | 0.97 |
|  | Thymogam + CSA +EPAG (3 months) | 2 | 14 | 29.90 | 51.97 |  |
| ATGAM Based | ATGAM + CSA (6 months) | 5 | 17.88 | 51.65 | 68.61* | 0.14 (compared to ORR with THYMOGAM + CSA at 6 months) |
| Anabolic steroids Only | Danazol or Stanozolol (3 months) | 1 | 11.05 | 8.72 | 19.77 |  |
|  | Danazol or Stanozolol (6 months) | 1 | 2.04 | 20.40 | 17.25* | 0.73 |
|  | Danazol or Stanozolol (12 months) | 1 | 4 | 16 | 20 | 0.79 |
| **Double-arm analysis – Response rates** | | | | | | |
| **Treatments** | | **No. of studies** (studies in which CR and PR data are separately given) | **CR (%)** | **PR (%)** | **Overall Response Rate (%)**  **Odds Ratio** | **p-values (Comparing ORRs from 3 to 6 months and 6 to 12 months between same treatment group)** |
| **THYMOGAM + CSA vs. ATGAM + CSA** | THYMOGAM + CSA (3 months) | 3 | 1.31 | 37.05 | 0.69 | 0.13 |
|  | ATGAM + CSA (3 months) | 3 | 3.72 | 42.11 |  |  |
|  | THYMOGAM + CSA (6 months) | 3 | 1.35 | 43.57 | 0.72 | 0.13 |
|  | ATGAM + CSA (6 months) | 3 | 19.25 | 36.02 |  |  |
|  | THYMOGAM + CSA (12 months) |  | NA | NA | 0.63 | 0.23 |
|  | ATGAM + CSA (12 months) |  | NA | NA |  |  |
| **THYMOGAM + CSA vs. CSA** | THYMOGAM + CSA (12 months) | 1 | 52.63 | 31.58 | 5.63 | 0.01 |
|  | CSA (12 months) | 1 | 8.11 | 40.54 |  |  |
| **CSA + Stanozolol vs CSA** | CSA + Stanozolol | 1 | 8.47 | 37.28 | 1.30 | 0.77 |
|  | CSA | 1 | 6.45 | 25.80 |  |  |
| **ATG + CSA vs. ATG + CAS + EPAG** | ATG + CSA | 1 | NA | NA | 0.67 | 0.50 |
|  | ATG + CSA + EPAG | 1 | NA | NA |  |  |

**ORR is not equal to the sum of CR and PR since one study did not report the stratified CR and PR data and was thus excluded from the CR and PR rate analysis

**ORR is not equal to the sum of CR and PR since the two studies did not report the stratified CR and PR data and were thus excluded from the CR and PR rate analysis.

**ORR is not equal to the sum of CR and PR since six studies did not report the stratified CR and PR data and were thus excluded from the CR and PR rate analysis.

**SF1.** Graphical representation of overall response rates for different treatment regimens at 3, 6, and 12 months.

**
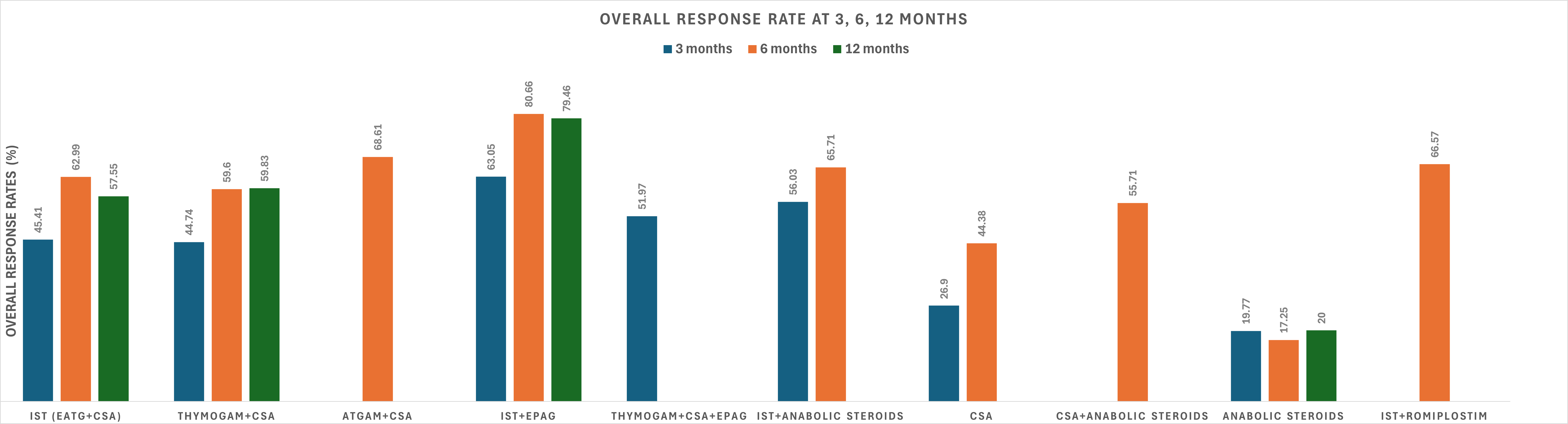
**

**SF2.** A comparison of the ORRs between patients administered with CSA monotherapy at 3 and 6 months.

**SF3.** A comparison of the ORRs between patients administered with anabolic steroid monotherapy at 3 and 6 months.

**SF4.** A comparison of the ORRs between patients administered with eATG combined with CSA and anabolic steroids.

**SF5.** A comparison of the ORRs between patients administered with eATG combined with CSA.

**SF6.** A comparison of the ORRs between patients administered CSA combined with anabolic steroids at 6 months.

**SF7.** A comparison of the ORRs between patients administered ATGAM combined with CSA.

**SF8.** A comparison of the ORRs between patients administered THYMOGAM combined with CSA and those with ATGAM plus CSA.

**SF9.** A comparison of the ORRs between patients administered eATG combined with CSA and EPAG.

**SF10.** A comparison of the ORRs between patients administered eATG combined with CSA and Romiplostim at 6 months.

**SF11.** A comparison of the ORRs between patients administered with anabolic steroid monotherapy at 6 and 12 months.

**SF12.** A comparison of the ORRs between patients administered with eATG plus CSA at 6 and 12 months.

**SF13.** A comparison of the ORRs between patients administered with THYMOGAM plus CSA at 6 and 12 months.

**SF14.** A comparison of the ORRs between patients administered with eATG plus CSA and EPAG at 6 and 12 months.

**DOUBLE ARM ANALYSIS**

**Overall Response Rate:**

**SF15.** A comparison of the ORRs between patients administered THYMOGAM combined with CSA and patients given with CSA only.

**Overall Survival**

**SF16.** Five years exponential survival curve for patients administered ATGAM combined with CSA.

**SF17.** Five years exponential survival curve for patients administered THYMOGAM combined with CSA.

**SF18:** Comparison of OS at 5 years among patients treated with ATGAM + CSA vs those treated with eATG + CSA

**SF19:** Comparison of OS at 5 years among patients treated with ATGAM + CSA vs those treated with eATG + CSA

**SF20.** Five years exponential survival curve for patients administered eATG combined with CSA and anabolic steroids.

**SF21.** Five years Event Free survival rate for patients administered eATG combined with CSA.****

**Publication Bias evaluation – Funnel plots**

**SF22.** Funnel chart showing the overall response rate between patients treated with THYMOGAM combined with CSA.

**Egger’s test**

| **Parameter** | **Value** |
| --- | --- |
| **beta1** | -1.52 |
| **SE of beta1** | 2.014 |
| **z** | -0.75 |
| **Prob > \|z\|** | 0.4515 |

The p-value (0.4515) is greater than 0.05, indicating no strong statistical evidence of publication bias based on Egger’s test.

**ST3:** Table showing the heterogeneity for different treatment groups at 3, 6, and 12 months.

| **Treatments** | | **Q** | **p-value** | **I2** |
| --- | --- | --- | --- | --- |
| **Equine ATG (eATG) Based** | eATG + CSA (3 months) | 41.36 | 0.00 | 83.11 |
|  | eATG + CSA (6 months) | 192.80 | 0.00 | 88.71 |
|  | eATG + CSA (12 months) | 8.16 | 0.23 | 27.49 |
|  | eATG + CSA+ EPAG (3 months) | 23.75 | 0.00 | 81.81 |
|  | eATG + CSA+ EPAG (6 months) | 1.88 | 0.60 | 0.00 |
|  | eATG + CSA + EPAG (12 months) | 0.86 | 0.35 | 0.00 |
|  | eATG + CSA + anabolic steroids (3 months) | 0.68 | 0.41 | 0.00 |
|  | eATG + CSA + anabolic steroids (6 months) | 1.67 | 0.43 | 0.00 |
|  | eATG + CSA + Romiplostim (6 months) | - | - | - |
| **CSA only** | CSA (3 months) | 16.08 | 0.00 | 86.51 |
|  | CSA (6 months) | 53 | 0.00 | 93.45 |
| **CSA + Other drugs** | CSA + anabolic steroids (6 months) | 3.57 | 0.06 | 72.01 |
| **Thymogam Based** | Thymogam+CSA (3 months) | 10.82 | 0.03 | 62.80 |
|  | Thymogam+CSA (6 months) | 30.43 | 0.00 | 74.30 |
|  | Thymogam+CSA (12 months) | 2.74 | 0.25 | 32.25 |
|  | Thymogam + CSA +EPAG (str3 months) | 6.82 | 0.01 | 85.34 |
| **ATGAM Based** | ATGAM + CSA (6 months) | 62.12 | 0.06 | 72.01 |
| **Other drugs Only** | Anabolic steroids Only (6 months) | 1.69 | 0.43 | 11.03 |

**LEAVE ONE OUT ANALYSIS – SINGLE ARM STUDIES**

**S4. LEAVE ONE OUT ANALYSIS – 3 MONTHS**

**S4.1 CSA Only**

| Omitted study | Effect size | [95% conf. interval] | | p-value |
| --- | --- | --- | --- | --- |
| Kumar R et al. 2004 | 34.075 | 7.428 | 60.722 | 0.012 |
| Varma S et al. 1999 | 23.657 | -4.7 | 52.013 | 0.102 |
| Shetty M et al. 2016 | 16.688 | 1.431 | 31.944 | 0.032 |
| Mandal PK et al. 2016 (3M) | 33.215 | 4.105 | 62.324 | 0.025 |
| theta | 26.899 | 4.755 | 49.044 | 0.017 |

**S4.2 eATG+CSA**

| **Omitted study** | **Effect size** | **[95% conf. interval]** | | **p-value** |
| --- | --- | --- | --- | --- |
| Agarwal et. al. 2015 | 45.90 | 35.10 | 56.71 | <0.0001 |
| Amalnath DS et al. 2019 | 43.32 | 33.17 | 53.46 | <0.0001 |
| Gupta V. et al. 2012 | 47.15 | 37.04 | 57.26 | <0.0001 |
| Shah S et al 2018 | 45.79 | 34.66 | 56.92 | <0.0001 |
| Jandial A et al 2017 | 45.13 | 34.21 | 56.06 | <0.0001 |
| Chattopadhyay et al. 2022 | 43.55 | 32.89 | 54.21 | <0.0001 |
| Shetty M et al. 2017 | 43.54 | 33.18 | 53.89 | <0.0001 |
| Ramzan et al. 2014 | 49.56 | 42.26 | 56.86 | <0.0001 |
| Choudhary DR et al. | 44.82 | 33.76 | 55.88 | <0.0001 |
| theta | 45.41 | 35.67 | 55.15 | <0.0001 |

**S4.3 eATG + CSA +EPAG**

| **Omitted study** | **Effect size** | **[95% conf. interval]** | | **p-value** |
| --- | --- | --- | --- | --- |
| Prusty SK et al. 2022 | 75.7 | 62.6 | 88.8 | <0.0001 |
| Radhakrishnan R et al. 2020 | 60.8 | 32.2 | 89.3 | <0.0001 |
| Radhika K.K 2021 | 58.1 | 30.7 | 85.6 | <0.0001 |
| Patel J et al. 2023 | 60.3 | 32.7 | 88.0 | <0.0001 |
| theta | 63.0 | 40.8 | 85.3 | <0.0001 |

**S4.4 Thymogam + CSA**

| **Omitted study** | **Effect size** | **[95% conf. interval]** | | **p-value** |
| --- | --- | --- | --- | --- |
| Agarwal et. al. 2015 | 45.60 | 32.69 | 58.51 | <0.0001 |
| Amalnath DS et al. 2019 | 40.26 | 33.14 | 47.39 | <0.0001 |
| Gupta V. et al. 2012 | 48.00 | 37.53 | 58.47 | <0.0001 |
| Shah S et al 2018 | 45.43 | 31.47 | 59.40 | <0.0001 |
| Jandial A et al 2017 | 44.08 | 30.85 | 57.30 | <0.0001 |
| theta | 44.74 | 34.17 | 55.31 | <0.0001 |

**S5. LEAVE ONE OUT ANALYSIS – 6 MONTHS**

**S5.1 ATGAM + CSA**

| **Omitted study** | **Effect size** | **[95% conf.interval]** | | **p-value** |
| --- | --- | --- | --- | --- |
| Samoon YJ et al. 2014 | 69.443 | 54.816 | 84.071 | <0.0001 |
| Nair V et al. 2014 | 64.994 | 53.297 | 76.691 | <0.0001 |
| Nair V et al 2011 | 65.112 | 53.482 | 76.742 | <0.0001 |
| Jena R et al. 2016 | 72.241 | 60.776 | 83.707 | <0.0001 |
| Mahapatra M et al. 2015 | 70.508 | 56.714 | 84.303 | <0.0001 |
| George B et al 2015 | 69.341 | 54.712 | 83.97 | <0.0001 |
| theta | 68.609 | 56.714 | 80.504 | <0.0001 |

**S5.2 CSA Only**

| **Omitted study** | **Effect size** | **[95% conf. interval]** | | **p-value** |
| --- | --- | --- | --- | --- |
| M Rai et al. 2001 | 44.982 | 20.851 | 69.112 | <0.0001 |
| Mahapatra M et al. 2015 | 47.643 | 23.349 | 71.937 | <0.0001 |
| Mandal PK et al. 2016 | 51.23 | 32.115 | 70.345 | <0.0001 |
| Korula A et al. 2015 | 42.037 | 16.939 | 67.136 | 0.001 |
| Gupta D et al. 2022 | 36.438 | 20.481 | 52.395 | <0.0001 |
| **theta** | **44.382** | **24.733** | **64.031** | **<0.0001** |

**S5.3 eATG + CSA**

| **Omitted study** | **Effect size** | **[95% conf.interval]** | | **p-value** |
| --- | --- | --- | --- | --- |
| Krishnan M et al. 2024 | 62.815 | 57.209 | 68.421 | <0.0001 |
| Agarwal et al. 2015 | 63.414 | 57.966 | 68.863 | <0.0001 |
| Amalnath DS 2019 | 62.733 | 57.168 | 68.298 | <0.0001 |
| Gupta V et al. 2012 | 64.044 | 58.918 | 69.171 | <0.0001 |
| Shah S. et al. 2018 | 63.65 | 58.242 | 69.059 | <0.0001 |
| Jandial A et al. 2017 | 63.063 | 57.534 | 68.592 | <0.0001 |
| Datta S et al. 2016 | 63.594 | 58.222 | 68.967 | <0.0001 |
| Delgado NDF, 2016 | 62.622 | 57.122 | 68.121 | <0.0001 |
| Jain PK et al. 2016 | 62.724 | 57.144 | 68.304 | <0.0001 |
| Gyi AA et al. 2020 | 62.466 | 57.031 | 67.901 | <0.0001 |
| Chattopadhyay et al 2022 | 62.413 | 56.899 | 67.927 | <0.0001 |
| Trehan et al. 2015 | 62.873 | 57.337 | 68.409 | <0.0001 |
| Nityanand S et al. 2015 | 63.359 | 57.855 | 68.863 | <0.0001 |
| Patel AB et al. 2015 | 63.479 | 58.094 | 68.863 | <0.0001 |
| Jagdish C et al 2008 | 63.663 | 58.34 | 68.986 | <0.0001 |
| Ramzan et al. 2014 | 62.904 | 57.393 | 68.415 | <0.0001 |
| Ganapule A et al. 2010 | 62.505 | 56.982 | 68.028 | <0.0001 |
| Ekbote VS et al. 2012 | 63.647 | 58.209 | 69.086 | <0.0001 |
| Samoon YJ et al. 2014 | 62.863 | 57.24 | 68.487 | <0.0001 |
| Nair V et al. 2014 | 61.945 | 56.83 | 67.06 | <0.0001 |
| Nair V. et al 2011 | 62.008 | 56.857 | 67.16 | <0.0001 |
| Jena R et al. 2016 | 63.603 | 58.168 | 69.038 | <0.0001 |
| Mahapatra M et al. 2015 | 63.145 | 57.569 | 68.721 | <0.0001 |
| U. Mani et al. 2010 | 62.562 | 57.135 | 67.988 | <0.0001 |
| George B et al. 2011 | 62.325 | 56.858 | 67.792 | <0.0001 |
| George B 2015 | 63.191 | 57.592 | 68.791 | <0.0001 |
| theta | 62.987 | 57.642 | 68.333 | <0.0001 |

**S5.4 eATG + CSA + Anabolic steroids**

| **Omitted Study** | **Effect Size** | **95% CI Lower** | **95% CI Upper** | **p-value** |
| --- | --- | --- | --- | --- |
| Kumar N et al. 2024 (Steroids) | 76.22 | 71.64 | 80.80 | <0.0001 |
| U. Mani et al. 2010 (Methylprednisolone) | 73.73 | 65.21 | 82.25 | <0.0001 |
| George B et al. 2011 (Methylprednisolone) | 69.81 | 56.53 | 83.09 | <0.0001 |
| **Pooled (theta)** | **75.40** | **71.00** | **79.80** | <0.0001 |

**S5.5 eATG + CSA +EPAG**

| **Omitted Study** | **Effect Size** | **95% CI Lower** | **95% CI Upper** | **p-value** |
| --- | --- | --- | --- | --- |
| Prusty SK et al. 2022 | 83.37 | 72.43 | 94.31 | <0.0001 |
| Karnati BV et al. 2023 | 81.49 | 74.73 | 88.24 | <0.0001 |
| Radhika K.K 2021 | 79.17 | 71.85 | 86.50 | <0.0001 |
| Patel J et al. 2023 | 80.06 | 73.25 | 86.86 | <0.0001 |
| **Pooled (theta)** | **80.66** | **74.14** | **87.18** | <0.0001 |

**S5.6 Anabolic Steroids only**

| **Omitted Study** | **Effect Size** | **95% CI Lower** | **95% CI Upper** | **p-value** |
| --- | --- | --- | --- | --- |
| Marwaha RK et al. 2004 (Stanozolol) | 10.65 | -3.56 | 24.86 | 0.142 |
| M Rai et al. 2001 (Stanozolol) | 21.07 | 10.42 | 31.72 | 0 |
| B.R. Agarwal et al. 1994 (Methylprednisolone) | 17.25 | 4.49 | 30.02 | 0.008 |
| **Pooled (theta)** | **17.32** | **7.45** | **27.20** | **0.001** |

**S5.7 THYMOGAM + CSA**

| **Omitted Study** | **Effect Size** | **95% CI Lower** | **95% CI Upper** | **p-value** |
| --- | --- | --- | --- | --- |
| Krishnan M et al. 2024 | 58.63 | 49.19 | 68.07 | <0.0001 |
| Agarwal et al. 2015 | 60.48 | 51.54 | 69.43 | <0.0001 |
| Amalnath DS 2019 | 58.46 | 49.29 | 67.63 | <0.0001 |
| Gupta V et al. 2012 | 62.19 | 55.09 | 69.29 | <0.0001 |
| Shah S. et al. 2018 | 61.12 | 52.33 | 69.91 | <0.0001 |
| Jandial A et al. 2017 | 59.49 | 50.24 | 68.74 | <0.0001 |
| Datta S et al. 2016 | 60.98 | 52.43 | 69.53 | <0.0001 |
| Delgado NDF, 2016 | 58.26 | 49.49 | 67.03 | <0.0001 |
| Jain PK et al. 2016 | 58.39 | 49.18 | 67.61 | <0.0001 |
| Gyi AA et al. 2020 | 57.90 | 49.60 | 66.19 | <0.0001 |
| **Pooled (theta)** | **59.60** | **51.30** | **67.90** | <0.0001 |

**S6. LEAVE ONE OUT ANALYSIS – 12 MONTHS**

**S6.1 eATG + CSA**

| **Omitted study** | **Effect size** | **[95% conf. interval]** | | **p-value** |
| --- | --- | --- | --- | --- |
| Krishnan M et al. 2024 | 54.63 | 48.23 | 61.04 | <0.0001 |
| Amalnath DS 2019 | 57.73 | 50.23 | 65.22 | <0.0001 |
| Shah S et al. 2018 | 57.34 | 49.22 | 65.46 | <0.0001 |
| Patel AB et al. 2015 | 58.58 | 52.29 | 64.88 | <0.0001 |
| Jagdish C et al 2008 | 58.71 | 52.42 | 65.00 | <0.0001 |
| Sharma R. et al. 2012 | 58.38 | 51.73 | 65.04 | <0.0001 |
| Ramzan et al. 2014 | 56.14 | 49.19 | 63.10 | <0.0001 |
| theta | 57.55 | 51.20 | 63.91 | <0.0001 |

**S6.2 Thymogam + CSA**

| **Omitted study** | **Effect size** | **[95% conf. interval]** | | **p-value** |
| --- | --- | --- | --- | --- |
| Krishnan & Amalnath 2024 | 55.56 | 47.44 | 63.67 | <0.0001 |
| Amalnath DS 2019 | 61.16 | 52.43 | 69.89 | <0.0001 |
| Shah S et al. 2018 | 61.37 | 51.69 | 71.05 | <0.0001 |
| theta | 59.83 | 52.74 | 66.92 | <0.0001 |

**LEAVE ONE OUT ANALYSIS – DOUBLE ARM STUDIES**

**S7. LEAVE ONE OUT ANALYSIS – 3 MONTHS (Double arm)**

**S7.1 THYMOGAM + CSA vs ATGAM + CSA**

| **Omitted Study** | **Risk Ratio** | **Lower CI** | **Upper CI** | **p-value** |
| --- | --- | --- | --- | --- |
| Sanyal C et al. 2022 | 0.88 | 0.64 | 1.19 | 0.41 |
| Dolai TK et al. 2015 | 0.83 | 0.65 | 1.07 | 0.16 |
| Dutta B. et al. 2021 | 0.87 | 0.65 | 1.15 | 0.32 |
| Jain A. 2024 | 0.74 | 0.54 | 1.00 | 0.05 |
| **Pooled (All studies)** | **0.83** | **0.65** | **1.06** | **0.14** |

**S7.2 Thymogam+CSA Vs ATGAM+CSA 6 months**

| **Omitted study** | **Risk ratio** | **[95% conf.interval]** | | **p-value** |
| --- | --- | --- | --- | --- |
| Malhotra et al. 2015 | 0.89 | 0.75 | 1.06 | 0.19 |
| Sanyal C et al. 2022 | 0.89 | 0.73 | 1.09 | 0.25 |
| Vidharti R et al. 2020 | 0.95 | 0.79 | 1.13 | 0.52 |
| Dolai TK et al. 2015 | 0.90 | 0.76 | 1.05 | 0.18 |
| Dutta B. et al 2021 | 0.86 | 0.72 | 1.03 | 0.10 |
| Jain A. 2024 | 0.85 | 0.71 | 1.02 | 0.09 |
| exp(theta) | 0.89 | 0.76 | 1.05 | 0.15 |

**META REGRESSION ANALYSIS**

**S8A. eATG+CSA**

| **Moderator** | **Coefficient** | **Std. Error** | **z-value** | **p-value** | **95% Confidence Interval** |
| --- | --- | --- | --- | --- | --- |
| Study Design (Retrospective) — ref = Prospective | -1.2699 | 7.7367 | -0.16 | 0.8696 | –16.4335 to 13.8937 |
| Intercept (_cons) | 59.2542 | 6.468 | 9.16 | <0.001 | 46.5772 to 71.9313 |

The pooled effect (intercept) is statistically significant (p < 0.001), indicating a consistent underlying effect across studies.
However, the moderator study design does not significantly influence the outcome (p = 0.8696), meaning the study design did not explain variation in effect sizes.

| **Parameter** | **Value** |
| --- | --- |
| Residual Heterogeneity (τ²) | 317.78 |
| I² (%) | 96.21 |
| H² | 26.37 |
| R-squared (%) | 0 |
| Qₑ (df = 27) | 3571.02 |
| p-value for Qₑ | <0.0001 |
| Qₘ (Moderator Test) | 0.027 (df = 1), p = 0.8696 |

There is substantial residual heterogeneity (I² = 96.2%, τ² = 317.78; Qₑ p < 0.0001), suggesting large between-study variability remains even after accounting for study design.
The model explains 0% of the heterogeneity (R² = 0.00%), indicating that other unmeasured study-level factors likely contribute to the observed differences across studies.

| **Moderator** | **Coefficient** | **Std. Error** | **z-value** | **p-value** | **95% Confidence Interval** |
| --- | --- | --- | --- | --- | --- |
| No. of patients (N) | -0.0296 | 0.0098 | -3.01 | 0.0026 | –0.0489 to –0.0103 |
| Intercept (_cons) | 63.8305 | 3.5317 | 18.07 | <0.001 | 56.9084 to 70.7526 |

| **Parameter** | **Value** |
| --- | --- |
| Residual Heterogeneity (τ²) | 221.24 |
| I² (%) | 91.92 |
| H² | 12.37 |
| R-squared (%) | 27.81 |
| Qₑ (df = 27) | 318.32 |
| p-value for Qₑ | <0.0001 |
| Qₘ (Moderator Test) | 9.06 (df = 1), p = 0.0026 |

The pooled effect (intercept) is statistically significant (p < 0.001), showing a consistent overall response rates across studies.
The moderator sample size (N) is a significant predictor (p = 0.0026), with a negative coefficient (–0.0296) indicating that larger studies tend to report lower ORR.

Although the model explains about 27.8% of the between-study heterogeneity (R² = 27.81%), substantial residual heterogeneity remains (I² = 91.9%, τ² = 221.24; Qₑ p < 0.0001).
This suggests that while study size accounts for part of the observed variation, other unmeasured study-level factors likely contribute to the remaining differences across studies.

| **Moderator** | **Coefficient** | **Std. Error** | **z-value** | **p-value** | **95% Confidence Interval** |
| --- | --- | --- | --- | --- | --- |
| Follow-Up 6 months (vs 3 months) | 6.7372 | 14.0025 | 0.48 | 0.6304 | –20.7071 to 34.1815 |
| Follow-Up 12 months (vs 3 months) | –3.9823 | 15.2452 | –0.26 | 0.7939 | –33.8623 to 25.8977 |
| Intercept (_cons) | 54.5592 | 13.3558 | 4.09 | <0.001 | 28.3824 to 80.7361 |

| **Parameter** | **Value** |
| --- | --- |
| Residual Heterogeneity (τ²) | 312.22 |
| I² (%) | 96.56 |
| H² | 29.04 |
| R-squared (%) | 0 |
| Qₑ (df = 26) | 3998.97 |
| p-value for Qₑ | <0.0001 |
| Qₘ (Moderator Test) | 1.69 (df = 2), p = 0.4297 |

The pooled effect (intercept) is statistically significant (p < 0.001), indicating a consistent underlying effect across studies. However, the moderator Follow-Up duration does not significantly influence the outcome (p = 0.630 for 6 months; p = 0.794 for 12 months), suggesting that follow-up length (6 or 12 months vs 3 months) did not explain variation in overall response rates.

There is substantial residual heterogeneity (I² = 96.6%, τ² = 312.22; Qₑ p < 0.0001), indicating considerable variation between studies that is not explained by follow-up duration.
The model explains 0% of the heterogeneity (R² = 0), suggesting that other study-level factors, beyond follow-up length, contribute to the observed variability.

**Multivariate meta regression:**

| **Moderator** | **Coefficient** | **Std. Error** | **z-value** | **p-value** | **95% Confidence Interval** |
| --- | --- | --- | --- | --- | --- |
| Intercept (_cons) | 56.0314 | 11.3558 | 4.93 | <0.001 | 33.7746 to 78.2883 |
| Study Design (Retrospective) ref - Prospective | 1.0221 | 6.3785 | 0.16 | 0.8727 | –11.4796 to 13.5237 |
| N | –0.0359 | 0.0096 | –3.73 | 0.0002 | –0.0547 to –0.0171 |
| FollowUp-6 months ( ref cat= 3 months) | 12.9701 | 11.6271 | 1.12 | 0.2646 | –9.8186 to 35.7589 |
| FollowUp-12 months ( ref cat= 3 months) | –3.6575 | 12.5463 | –0.29 | 0.7707 | –28.2479 to 20.9329 |

| Residual Heterogeneity (τ²) | 193.44 |
| --- | --- |
| I² (%) | 90.35 |
| H² | 10.36 |
| R-squared (%) | 36.88 |
| Qₑ (df = 24) | 225.71 |
| p-value for Qₑ | <0.001 |
| Qₘ (Moderator Test) | 16.41 (df = 4), p = 0.0025 |

The pooled effect (intercept) is statistically significant (p < 0.001), indicating a consistent underlying overall response rate across studies.
Among the moderators, sample size (N) shows a significant negative association with the outcome (β = –0.0359, p = 0.0002), suggesting that larger studies tend to report a lower response rate.

Other moderators, Study Design, Follow Up, do not significantly influence the outcome (all p > 0.05), indicating they do not explain additional variation in overall response rates.

There is significant residual heterogeneity (I² = 90.35%, τ² = 193.44; Qₑ p < 0.001), but the model explains 36.9% of the between-study variance, meaning the included moderators account for a moderate portion of variability across studies.

**S8B: THYMOGAM+CSA**

**Univariate meta regression**

| **Moderator** | **Coefficient** | **Std. Error** | **z-value** | **p-value** | **95% Confidence Interval** |
| --- | --- | --- | --- | --- | --- |
| **Study Design(Retrospective) ref cat = prospective** | 7.2201 | 8.9154 | 0.81 | 0.418 | –10.2538 to 24.6941 |
| **Intercept (_cons)** | 52.37 | 7.4595 | 7.02 | <0.001 | 37.7495 to 66.9903 |

The pooled effect is significant (p < 0.001), indicating a consistent effect across studies. Study design does not significantly influence outcomes (p = 0.418).

| **Parameter** | **Value** |
| --- | --- |
| **Residual Heterogeneity (τ²)** | 111.27 |
| **I² (%)** | 69.1 |
| **H²** | 3.24 |
| **R-squared (%)** | 0 |
| **Qₑ (df = 8)** | 24.05 |
| **p-value for Qₑ** | 0.0023 |
| **Qₘ (Moderator Test)** | 0.66 (df = 1), p = 0.418 |

There is significant residual heterogeneity (I² = 69.1%, τ² = 111.27; Qₑ p = 0.0023), suggesting that substantial between-study variation remains after accounting for *Study Design*. The model explains 0% of the variance, indicating that other unmeasured factors may contribute to differences among studies.

| **Moderator** | **Coefficient** | **Std. Error** | **z-value** | **p-value** | **95% Confidence Interval** |
| --- | --- | --- | --- | --- | --- |
| Intercept (_cons) | 55.9322 | 7.2109 | 7.76 | <0.001 | 41.7990 to 70.0654 |
| N | 0.0234 | 0.0931 | 0.25 | 0.8013 | –0.1591 to 0.2060 |

The pooled effect (intercept) is statistically significant (p < 0.001), indicating a consistent underlying effect across studies. The moderator **N** does not significantly affect the outcome (p = 0.8013), suggesting it does not explain variation in effect sizes.

| **Parameter** | **Value** |
| --- | --- |
| Residual Heterogeneity (τ²) | 122.51 |
| I² (%) | 71.32 |
| H² | 3.49 |
| R-squared (%) | 0 |
| Qₑ (df = 8) | 24.61 |
| p-value for Qₑ | 0.0018 |
| Qₘ (Moderator Test) | 0.0633 (df = 1), p = 0.8013 |

There is significant residual heterogeneity (I² = 71.32%, τ² = 122.51; Qₑ p = 0.0018), suggesting that substantial between-study variation remains after accounting for the moderator N. The model explains 0% of the variance, indicating that other unmeasured factors may contribute to the observed differences among studies.

| **Moderator** | **Coefficient** | **Std. Error** | **z-value** | **p-value** | **95% Confidence Interval** |
| --- | --- | --- | --- | --- | --- |
| Intercept (_cons) | 58.9231 | 5.1797 | 11.38 | <0.001 | 48.7711 to 69.0750 |
| Follow-Up12 months (Vs 6 months) | –4.1903 | 8.6342 | –0.49 | 0.6275 | –21.1131 to 12.7325 |

The pooled effect (intercept) is statistically significant (p < 0.001), indicating a consistent underlying effect across studies. The moderator Follow-Up does not significantly affect the outcome (p = 0.6275), suggesting it does not explain variation in effect sizes.

| **Parameter** | **Value** |
| --- | --- |
| Residual Heterogeneity (τ²) | 115.95 |
| I² (%) | 70.91 |
| H² | 3.44 |
| R-squared (%) | 0 |
| Qₑ (df = 8) | 23.45 |
| p-value for Qₑ | 0.0028 |
| Qₘ (Moderator Test) | 0.2355 (df = 1), p = 0.6275 |

There is significant residual heterogeneity (I² = 70.91%, τ² = 115.95; Qₑ p = 0.0028), suggesting that substantial between-study variation remains after accounting for the moderator Follow Up. The model explains 0% of the variance, implying that other unmeasured study-level factors may contribute to the observed heterogeneity.

**Note:-**

- *For the eATG + CSA group, there are 29 studies and 3 covariates, which meets the general guideline of having about 10 studies per covariate. This provides sufficient power and stability for model estimation. Therefore, both univariate and multivariate meta-regression analyses are reported to assess the independent and combined effects of the moderators.*
- *For the THYMOGAM + CSA subgroup, the number of available studies is insufficient to support multivariate meta-regression. Since reliable estimation typically requires at least 10 studies per covariate, including multiple predictors would lead to unstable and unreliable results. Therefore, only univariate meta-regression results are reported for this group to maintain statistical validity and interpretability.*

**SAFETY ANALYSIS IN DIFFERENT TREATMENT GROUPS**

**ST4:** Summary of Adverse Events (Grades 1–5) reported across studies by treatment regimen in AA patients.

| **GRADE 1** | **GRADE 2** | **GRADE 3** | **GRADE 4** | **GRADE 5** |
| --- | --- | --- | --- | --- |
| **eATG + CSA (24 studies- 1922 patients)** | | | | |
| Gingival Hyperplasia – 37 | Hypertension – 160 | Pneumonia - 5 | Intracranial Hemorrhage – 7 | Death - 225 |
| Gum Hypertrophy – 62 | Acute renal failure – 3 | Febrile Neutropenia – 52 | Pulmonary Edema - 1 |  |
| Renal Dysfunction - 2 | Seizure - 1 | Hemolytic anemia - 1 |  |  |
| Hepatotoxicity - 3 | Renal dysfunction - 4 | Avascular necrosis - 47 |  |  |
| Hypertrichosis - 20 | Serum sickness – 183 |  |  |  |
| Diabetes Mellitus - 21 | Non-fatal Febrile Neutropenia - 6 |  |  |  |
| Fever - 25 | Acute Kidney Injury - 57 |  |  |  |
| Tremor – 4 | Vasculitis - 1 |  |  |  |
| Sweating - 3 |  |  |  |  |
| Vomiting, Allergic Urticaria, Deranged LFT, Bilateral pedal edema, Joint pain, chest pain, Tachycardia, Hyperglycemia– 2 each |  |  |  |  |
| Elevation of Liver enzyme, Abscess in neck, Papular rash, swelling on face, Weight gain, Gingivitis, Bilateral pleural effusion, Burning of eyes , Shivering, Acute suppurative otitis media – 1 each |  |  |  |  |
| **Thymogam + CSA (9 studies – 548 patients)** | | | | |
| Gum Hypertrophy – 48 | Hypertension – 18 | Pneumonia - 5 | Intracranial Hemorrhage – 7 | Death - 76 |
| Fever - 13 | Acute renal failure – 3 | Febrile Neutropenia – 52 | Pulmonary Edema - 1 |  |
| Tremor - 4 | Seizure - 1 | Hemolytic anemia - 1 |  |  |
| Sweating - 3 | Serum sickness – 24 |  |  |  |
| Vomiting, Allergic Urticaria, Bilateral pedal edema, Joint pain, Hyperglycemia, chest pain, tachycardia – 2 each | Non-fatal Febrile Neutropenia - 6 |  |  |  |
| Abscess in neck, Papular rash, swelling on face, Weight gain, Gingivitis, Bilateral pleural effusion, Burning of eyes , Shivering - 1 each | Acute Kidney Injury - 3 |  |  |  |
|  | Vasculitis - 1 |  |  |  |
| **ATGAM + CSA (4 studies – 360 patients)** | | | | |
| Fever - 17 | Rigor - 4 | Lower respiratory tract infection - 10 |  | Death - 25 |
|  | Serum sickness – 40 |  |  |  |
|  | Urticaria - 4 |  |  |  |
|  | Myalgia - 5 |  |  |  |
|  | Mucocutaneous bleed - 8 |  |  |  |
|  | Arthralgia - 18 |  |  |  |
|  | Upper respiratory tract infection - 5 |  |  |  |
| **eATG + CSA + EPAG (6 studies – 188 patients)** | | | | |
| Renal Dysfunction – 3 | Hypotension - 4 | Hyperbilirubinemia -21 | Transaminitis - 12 | Death - 27 |
| **THYMOGAM + CSA + anabolic steroids (2 studies – 107 patients)** | | | | |
| Renal Dysfunction – 3 |  | Hyperbilirubinemia -7 | Transaminitis - 8 | Death - 19 |
| **eATG + CSA + anabolic steroids (3 studies – 519 patients)** | | | | |
|  | Serum sickness - 1 | Herpes zoster infection - 1 |  | Death - 12 |
|  | Jaundice - 23 | Fungal pneumonia - 2 |  |  |
| **CSA Only (3 studies – 92 patients)** | | | | |
| Gum hypertrophy – 9 |  |  |  | Death - 3 |
| Gingival Hyperplasia - 2 |  |  |  |  |
| Renal Dysfunction - 6 |  |  |  |  |
| **Anabolic steroids (2 studies- 74 patients)** | | | | |
|  |  |  |  | Death - 15 |
| **ATG + CSA + Romiplostim (1 study, 12 patients)** | | | | |
| Gum hypertrophy - 1 | Serum sickness - 1 | Febrile Neutropenia - 3 |  | Death - 2 |
|  | Hypertension - 1 | Intracranial Haemorrhage - 1 |  |  |

**ST4: Risk of bias/quality assessment using New Castle Ottawa scale for observational studies**

| **Study Name** | **Selection** | | | | **Comparability** | **Outcome** | | |
| --- | --- | --- | --- | --- | --- | --- | --- | --- |
|  | **Representativeness of the exposed cohort** | **Selection of the non-exposed cohort** | **Ascertainment of exposure** | **Outcome not present at start** | **Comparability of cohorts** | **Assessment of outcome** | **Follow-up long enough** | **Adequacy of follow-up** |
| Sanyal C 2022 | Low | NA | Low | Low | Unclear | Low | Low | High |
| Dolai TK 2015 | Low | NA | Low | Low | Unclear | Low | Low | Low |
| Dutta B 2021 | Low | NA | Low | Low | Low | Low | Low | Low |
| Jain A 2024 | Low | NA | Low | Low | Low | Low | Low | High |
| Malhotra 2015 | Low | NA | Low | Low | Low | Low | Low | High |
| Rishu Vidharti 2020 | Low | NA | Low | Low | Unclear | Unclear | Low | Low |
| Monisha 2020 | Low | NA | Low | Low | Unclear | Unclear | Low | Low |
| Kotha A 2010 | Unclear | NA | Low | Low | Unclear | Unclear | High | Low |
| Chandrakala 2014 | Low | NA | Low | Low | Unclear | Unclear | Low | Low |
| Mantri S 2017 | Low | NA | Low | Low | Unclear | Unclear | Low | Low |


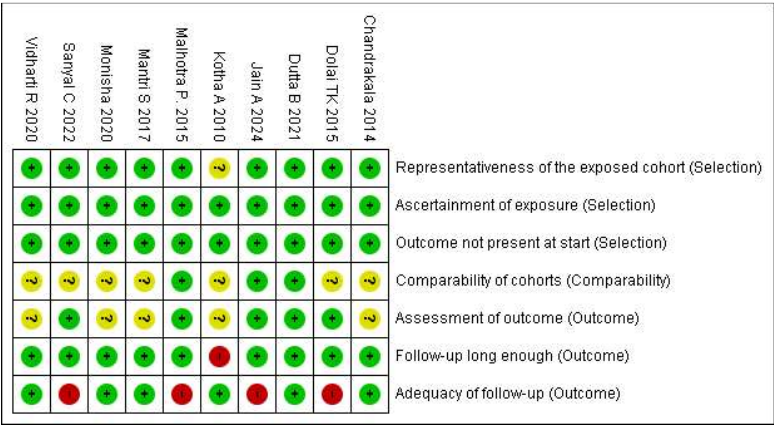


**Fig:** A comparison of the ORRs between THYMOGAM plus CSA between studies conducted in India and Cuba, and Myanmar at 6 months.
